# Lean-water hydrogel electrolyte for zinc ion batteries

**DOI:** 10.1038/s41467-023-39634-8

**Published:** 2023-07-01

**Authors:** Yanbo Wang, Qing Li, Hu Hong, Shuo Yang, Rong Zhang, Xiaoqi Wang, Xu Jin, Bo Xiong, Shengchi Bai, Chunyi Zhi

**Affiliations:** 1 Department of Materials Science and Engineering, City University of Hong Kong, 83 Tat Chee Avenue, Kowloon, Hong Kong 999077 China; 2 Research Institute of Petroleum Exploration & Development (RIPED), PetroChina Research Center of New Energy, No. 20 Xueyuan Road, Haidian District, Beijing, 100083 China; 3 Centre for Functional Photonics, City University of Hong Kong, Kowloon, Hong Kong 999077 China; 4 Hong Kong Institute for Advanced Study, City University of Hong Kong, Kowloon, Hong Kong 999077 China

**Keywords:** Batteries, Batteries, Polymer chemistry, Batteries

## Abstract

Solid polymer electrolytes (SPEs) and hydrogel electrolytes were developed as electrolytes for zinc ion batteries (ZIBs). Hydrogels can retain water molecules and provide high ionic conductivities; however, they contain many free water molecules, inevitably causing side reactions on the zinc anode. SPEs can enhance the stability of anodes, but they typically possess low ionic conductivities and result in high impedance. Here, we develop a lean water hydrogel electrolyte, aiming to balance ion transfer, anode stability, electrochemical stability window and resistance. This hydrogel is equipped with a molecular lubrication mechanism to ensure fast ion transportation. Additionally, this design leads to a widened electrochemical stability window and highly reversible zinc plating/ stripping. The full cell shows excellent cycling stability and capacity retentions at high and low current rates, respectively. Moreover, superior adhesion ability can be achieved, meeting the needs of flexible devices.

## Abstract (web summary)

Excess water in hydrogel-based zinc ion batteries causes side reactions, but reduced water content results in low conductivities. Here, authors develop a lean-water hydrogel based on molecular lubrication mechanism for fast ion transportation, extended stability, and reversible Zinc plating/stripping.

## Introduction

Zinc ion batteries (ZIBs), owing to their high theoretical capacity (820 mAh g^−1^), safety and reliability, are promising for a variety of applications^1–3^. However, the parasitic hydrogen evolution reaction (HER) and irreversible loss of Zn^2+^ from the electrolyte can significantly decrease the coulombic efficiency and stability of aqueous ZIBs^4,5^. Additionally, high current rates are usually performed in ZIBs to investigate the cycle-life duration, but the results are less meaningful on realistic estimations of battery lifetimes^6^. To obtain solid-state/quasi-solid-state ZIBs, solid polymer electrolytes (SPEs) and hydrogel electrolytes (HPEs) were developed^7^. HPEs (polyacrylamide (PAM), poly (acrylic acid) (PAA), etc.) possess high ionic conductivities in the range of 10^−3^ to 10^−2^ S cm^-1^^8,9^. However, a large amount of water (typically >80 wt% based on the method of (m_t_ − m_0_)/m_t_ × 100%, where m_t_ and m_0_ are the masses of swollen and dried samples, respectively) is present in HPEs, leading to a narrow electrochemical stability window. Moreover, HPEs cannot fundamentally eliminate problems of the anode, such as HER, dendrites, and corrosion^10^.

On the other hand, due to their liquid-free property and high chemical stability, SPEs are a promising approach to achieve a highly stable Zn anode and wide electrochemical stability window. However, SPEs usually can only achieve low ionic conductivities (10^−4^ to 10^−6^ S cm^−1^) as well as high interfacial resistances; as a result, the fabricated batteries exhibit poor rate ability and high resistance^7,11–13^.

**Fig. 1:**
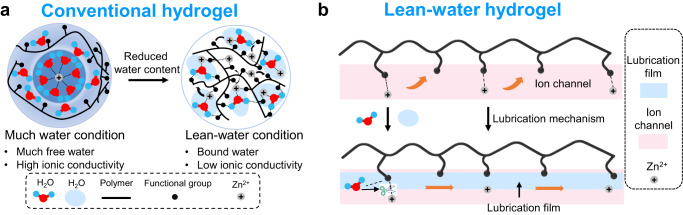
Schematic illustration of the ionic transportation mechanism of hydrogels. **a** Conventional hydrogel electrolyte under much and lean-water conditions (confined Zn^2+^). **b** The expected ionic transportation mechanism for hydrogels under lean-water conditions: formation of ion transportation channels and lubrication mechanism.

Considering the merits and shortcomings of HPEs and SPEs, lean-water hydrogels may provide an important balance between the two; the advantages of HPEs and SPEs, including their wide electrochemical stability window, dendrite-free and gas-evolution-free anodes and ability to perform rapid ionic transportation, could be retained. However, for conventional hydrogels, when the water content is dramatically reduced, the free water molecules are suppressed, retaining the strongly bound water. The Zn^2+^ is intensively confined by the hydrophilic groups in polymer matrices, resulting in a dramatically decreased ionic conductivity (Fig. 1a)^14–19^.

If a polymer matrix can be designed with specific ionic channels to ensure that ionic dissociation and conduction occur, then the transportation of ions may not largely rely on water molecules, and the loss of ionic conductivity should be effectively compensated (Fig. 1b). In addition, as the charge density of divalent Zn^2+^ is higher than that of Li^+^, their interaction with the polymer matrix is more intensive, and ionic diffusion is rather difficult^13^. In tribology studies, lubrication is an effective way to reduce resistance. The strong interaction of the lubricants with the friction surfaces and the lubrication films at the molecular scale is the main methodology for achieving excellent lubricating properties and reduced resistances^20–22^. Among various lubricants, water molecules are highly effective; compared to nonassociating liquids, such as oil and organic solvents, water molecules exhibit a low viscosity and good fluidity^23,24^. Based on this strategy, a potential approach is to enhance the interactions between the water molecules and the functional groups in the polymer matrix (Fig. 1b). In this case, the lean-water molecules can act as lubricants to weaken the intermolecular polymer-polymer and polymer-ion interactions. This will result in intensive chain segmental motions of the polymer matrix and improved dissociation of ions and their mobility. Therefore, taking advantage of lubrication effects is a promising strategy to develop lean-water hydrogel electrolytes equipped with the merits of both HPEs and SPEs.

In this study, we select a polymeric zwitterion (PZI) as a polymer skeleton in which the sulfonate terminals combine the hydrophilic and zincophilic properties and a zinc salt functions as the coordination unit. The sulfonate anion functions as a hydrogen bond acceptor, which can be lubricated by water molecules to promote the dissociation of zinc ions as well as enhance the electrochemical stability of water molecules. The designed lean-water hydrogel electrolyte delivers a widened voltage window and ionic conductivity of 2.6 × 10^−3^ S cm^−1^ under lean-water content (20 wt%). The full cell exhibits negligible gas evolution and excellent cycling performance of 4000 cycles and 1500 cycles at 5 C and 1 C, respectively. In addition, the lean-water hydrogel electrolyte exhibits high adhesion to electrodes, providing firm interfacial in flexible ZIBs. The design of lean-water hydrogel electrolytes provides an important balance between HPEs and SPEs to gain both high stability and ionic conductivity.

## Results

### Lean-water hydrogel electrolyte

**Fig. 2:**
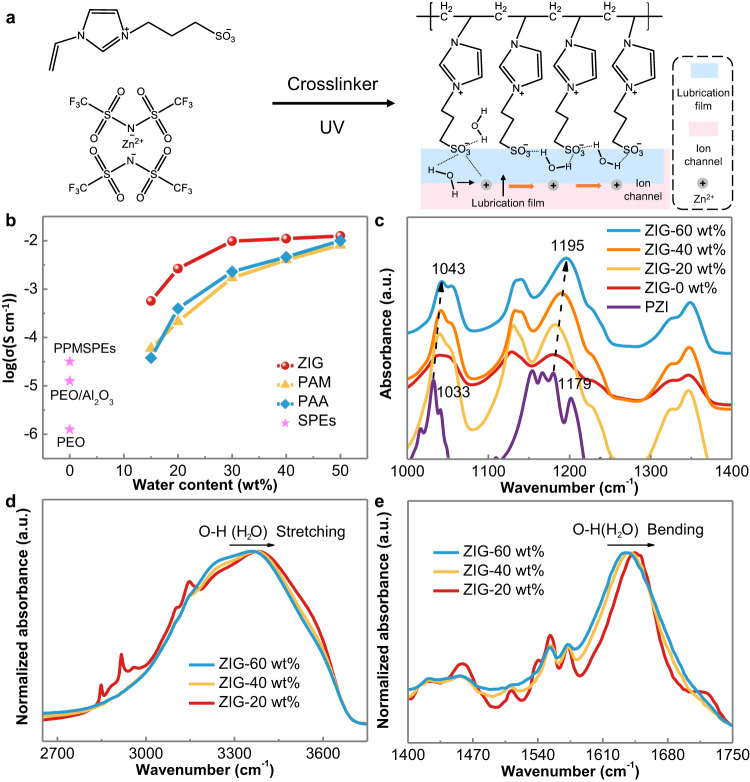
Chemical structure of ZIG and FTIR spectra of the synthesized hydrogel. **a** Synthesis route and the functional mechanism of ZIG. **b** Ionic conductivities of different hydrogel electrolytes with different water contents and SPEs. **c** FTIR spectra of PZI and ZIGs with different water contents (0, 20, 40, 60 wt%). **d** Stretching region of the normalized FTIR spectra of ZIGs with different water contents (20, 40, 60 wt%). **e** Bending region of the normalized FTIR spectra of ZIGs with different water contents (20, 40, 60 wt%).

The selected precursors, fabrication and structure of the zwitterion hydrogel electrolyte (ZIG) are shown in Fig. 2a. ZIG was prepared through two-step reactions, and the detailed procedures are described in the supporting information (SI). In brief, the monomer of 3-(1-vinyl-3-imidazolio) propanesulfonate (VIPS) was synthesized^25^. Subsequently, the precursor of the ZIG was prepared by mixing a certain mole ratio of VIPS, zinc di[bis(trifluoromethylsulfonyl)imide] (Zn (TFSI)_2_), poly(ethylene glycol) dimethacrylate (PEGDMA) and 2-hydroxy-4’-(2-hydroxyethoxy)-2-methylpropiophenone as the skeleton, salt, cross-linker and photoinitiator, respectively, in aqueous solution. After UV-induced polymerization, a facile and flexible polymer film could be obtained. The size and thickness could be easily controlled by homemade glass moulds. The hydrogel structure was investigated and confirmed by Fourier transform infrared (FTIR) spectroscopy and nuclear magnetic resonance (NMR), as shown in Supplementary Figs. 1–3, verifying the successful reactions^25^. The results of thermogravimetric analysis (TGA) illustrated that ZIG remained thermally stable until 250 °C (Supplementary Fig. 4).

ZIG is a typical polyzwitterionic material with positively and negatively charged groups as repeating units. After mixing with proportional Zn(TFSI)_2_, ion migration channels could be constructed for Zn^2+^ due to the generation of ion pairs on the basis of electrostatic interactions and acid and base principles^26–28^. Furthermore, optical images were obtained before and after the zinc salt was mixed, revealing the change from a solid-state mixture of VIPS and Zn(TFSI)_2_ to a liquid state, manifesting the zincophilic properties of the polymer framework (Supplementary Fig. 5). The sulfonate terminals in the polymeric framework could function as Zn^2+^-rich channels for Zn^2+^ transportation^25,28,29^. Then, the small amount of water molecules (lean-water content) could interact with the charged groups and form molecular lubrication films to reduce the interaction between the Zn^2+^-polymer chain and polymer chain-polymer chain, promoting the separation and transportation of ion pairs and improving ionic transportation (Fig. 2a). The interaction could be ascribed to the strong affinity of PZI for water. The sulfur atom in the PZI is surrounded by three oxygen atoms, which not only carry a negative charge but also contain lone pairs of electrons for hydrogen bonding and ionic solvation with water^30,31^.

The ionic conductivities of the synthesized hydrogels with different water contents were determined by electrochemical impedance spectroscopy (EIS). As shown in Fig. 2b and Supplementary Fig. 6, ZIG, PAM and PAA display similar ionic conductivities (approximately 8.0 × 10^−3^ S cm^−1^) at 50 wt% water content. However, when the water content decreased, the ionic conductivities of ZIG remained higher than those of PAM and PAA. When the water content is below 30 wt%, the ionic conductivity of ZIG can be approximately one order of magnitude higher than those of PAM and PAA. With a 20 wt% water content, the ionic conductivity of ZIG is approximately 2.6 × 10^−3^ S cm^−1^, in contrast to the 2.1 × 10^−4^ S cm^−1^ of PAM and 3.9 × 10^−4^ S cm^−1^ of PAA. The ionic conductivity is highly comparable with that of other reported electrolytes for lithium-ion batteries and aqueous batteries (Supplementary Fig. 7). Therefore, ZIG with 20 wt% water content (ZIG-20 wt%) exhibits a good balance and possesses much higher ionic conductivity than that of SPEs and other hydrogels under the same water content. This can support high-performance expression of ZIBs and an excellent rate ability. On the other hand, with lean water content, a wide electrochemical stability window is expected and a variety of side reactions of the Zn anode should be effectively suppressed.

Different characterizations were conducted to reveal the mechanism of the achieved impressive ionic conductivity (10^−3^ S cm^−1^) of ZIG under lean-water conditions. The interactions among PZI, Zn^2+^, and water molecules were confirmed by FTIR (Fig. 2c). The two absorption peaks at 1033 and 1179 cm^−1^ were attributed to the stretching vibration of S = O in the sulfate groups^32,33^. When Zn^2+^ is introduced, both peaks blueshift to 1038 and 1181 cm^−1^, evidencing the intermolecular electrostatic interactions between Zn^2+^ and SO_3_^−^. After water was added, these two peaks shifted to higher wavenumbers. This is because water molecules surrounding the SO_3_^−^ groups can function as lubricants and reduce the binding force between Zn^2+^ and SO_3_^−^. Differential scanning calorimetry (DSC) was further used to study the function of water in the ZIG (Supplementary Fig. 8). Introducing water molecules clearly affects the glass transition temperature (T_g_) of ZIG. When no water was introduced, the highest T_g_ of 38 °C was obtained, indicating a rigid network structure and unfavorable ionic transportation environment. With the increase in water content to 20 wt%, the T_g_ decreases dramatically to −47 °C. This is because the zwitterionic surfaces could be hydrated by electrostatic interactions and hydrogen bonds, forming a water lubrication layer. These interactions between PZI and water (without zinc salt) could be verified by the similar shifts observed for vibrations of S = O and O-H as ZIG in FTIR (Supplementary Fig. 9). The water layer can significantly suppress the interaction between polymer chains, which leads to enhanced chain segmental motions. Furthermore, ^67^Zn NMR was performed to investigate the interaction between water and Zn^2+^. As shown in Supplementary Fig. 10, for the Zn(TFSI)_2_ solution without the polymer skeleton, the chemical shift is approximately 0 ppm. After the polymer skeleton was introduced, the chemical shift increased to 0.5 ppm with a widened peak, indicating a varied Zn^2+^ chemical environment was induced by the interaction with sulfonate terminals. When the water content was decreased to 20 wt%, the signal shifted to −0.5 ppm, which is due to the interaction between water molecules and Zn^2+^. In the presence of water molecules, Zn^2+^ ions are solvated by water molecules to produce a series of deprotonated compounds, and an acidic solution with a change in pH can be obtained. As shown in Supplementary Fig. 11, the pH values increase from ~3 for ZIG-60 wt% to ~7 for ZIG-10 wt% with decreasing water content, indicating a remarkably weakened hydrolysis effect. At low water contents, water molecules tend to interact with SO_3−_ to form lubrication layers, decreasing the water content in the vicinity of Zn^2+^ and diminishing the Zn^2+^-water interaction. Therefore, Zn^2+^ could move as depicted in Fig. 2a. In addition, the environment of the TFSI^−^ anion was further investigated. As shown in Supplementary Fig. 12, the peak at 1575 cm^−1^ of PZI is assigned to the C = N in imidazole. After TFSI^-^ was introduced, the peak shifted, suggesting an interaction between the cationic imidazole and anion. Notably, the peak maintains its position with the change in water content. Thus, it is believed that the TFSI^-^ anions couple with cationic imidazole in the polymer framework. Additionally, the Raman vibration at ~745 cm^−1^ is assigned to the S-N-S bending vibration in TFSI^−^ (Supplementary Fig. 13). The vibration remains unchanged at different water contents, suggesting that water molecules have less influence on the anion environment. These observations clearly demonstrate that the lean-water strategy is feasible and that the lubrication mechanism is responsible for the separation and transportation of Zn^2+^ in the lean-water environment^34^. Additionally, the O-H stretching and bending vibration modes are displayed in Fig. 2d, e, respectively. The typical O-H stretching vibration (3200−3400 cm^−1^) and O-H bending vibration (1638 cm^−1^) of water molecules shift to higher wavenumbers with decreasing water content^16,18^. The blueshift was caused by the interaction between H in water molecules and O in SO_3_^−^. SO_3_^−^ groups possess a high electron density, which can weaken the hydrogen bonds between water‒water. The strengthened O-H covalent bonds could increase the electrochemical stability of water and suppress its decomposition in ZIG^35^.

### Electrochemical characterizations of the Zn metal electrode with the lean-water hydrogel electrolyte

**Fig. 3:**
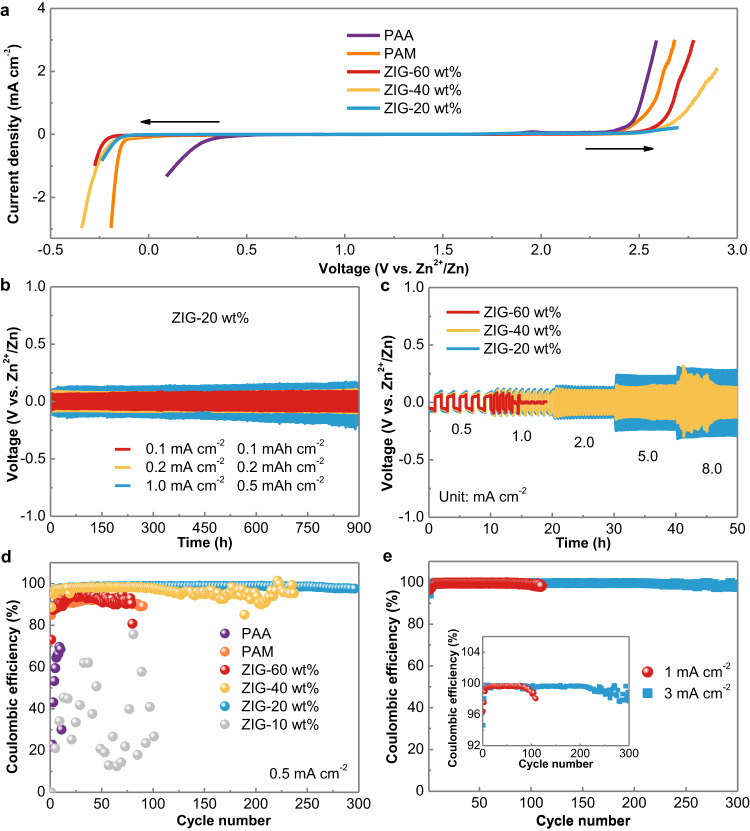
Electrochemical stability windows of different electrolytes and stability of the Zn anode. **a** Linear sweep voltammetry of ZIG (20, 40, 60 wt% water content), PAM and PAA (conventional HPEs). **b** Galvanostatic Zn plating/stripping in Zn | |Zn symmetrical cells with ZIG-20 wt% electrolyte with different current densities and different plating capacities. **c** Rate performance at different current densities with an areal capacity of 0.5 mAh cm^−2^. **d** Coulombic efficiency of Zn deposition based on Zn | |Cu cells with different electrolytes. **e** Coulombic efficiencies of Zn deposition based on the Zn | |Cu cell with ZIG-20 wt% electrolyte.

The electrochemical stability window of the developed hydrogels was further evaluated with linear sweep voltammetry (LSV). The overall stability window of the ZIG significantly expanded with the decrease in water content from 60 wt% to 20 wt% (Fig. 3a, Supplementary Fig. 14), which is much wider than those of PAA and PAM (conventional HPEs). The improvement is mainly ascribed to the enhanced O-H covalent bond of water with decreasing water content. Furthermore, gas generation based on ZIG-20 wt%, PAA and PAM hydrogels as electrolytes was monitored in situ with Zn | |Zn symmetric batteries at a current density of 1 mA cm^−2^ and capacity of 0.5 mAh cm^−2^ (Supplementary Fig. 15). The PAA hydrogel showed the highest gas pressure, which was approximately ten times higher than that of the PAM hydrogel after 60 mins. This is mainly because PAA is more acidic than PAM. In contrast, the gas evolution was negligible for ZIG-20 wt%, demonstrating the successful suppression of side reactions through our lean-water strategy. Additionally, an optical microscope was used to obtain in situ photographs of the plated Zn anodes. As shown in Supplementary Fig. 16, for the PAA hydrogel electrolyte, obvious bubbles resulting from the gas evolution reaction were observed after 1 min, and they continued growing. A similar phenomenon was observed for the PAM hydrogel electrolyte, as small bubbles formed after 5 min. Comparatively, no bubbles were found on the surface of Zn with the ZIG-20 wt% electrolyte even after 15 min. These results confirm that the ZIG-20 wt% electrolyte exhibits an ability to inhibit gas evolution.

Then, the reversibility and stability of the Zn | |Zn symmetric cells based on different hydrogel electrolytes were evaluated. As shown in Fig. 3b, different current densities and plating capacities were inspected to clarify the reversible Zn deposition/dissolution process. For ZIG-20 wt%, approximately 900 h of stable plating/stripping was attained at current densities of 0.1 mA cm^−2^ and 0.2 mA cm^−2^. Even when the current density increased to 1.0 mA cm^−2^, an excellent lifespan could be achieved, which was ascribed to the uniform Zn deposition process and stable interface, and after cycling at a current density of 1.0 mA cm^−2^, the Zn metal showed a smooth, dense and dendrite-free surface morphology (Supplementary Fig. 17). Correspondingly, the X-ray diffraction (XRD) pattern further confirmed that no zinc oxides or hydroxides (Supplementary Fig. 18) were produced. In sharp contrast, the result of Zn deposition/dissolution with PAA and PAM hydrogels revealed large increases in voltage hysteresis at a current density of 1.0 mA cm^−2^, as shown in Supplementary Fig. 19. This occurred due to the corrosion of metallic Zn and the formation of intensified Zn dendrite, as the description of morphologies after cycles in Supplementary Fig. 17. The corresponding byproducts of zinc oxides or hydroxides after plating/stripping cycles were also confirmed by XRD (Supplementary Fig. 18). More importantly, PAA and PAM with the same water content (20 wt%) as ZIG-20 wt% were also tested in Zn symmetric cells. They showed a strong voltage hysteresis of 2.0 V and 0.4 V even at a low current density of 0.2 mA cm^−2^ (Supplementary Fig. 20), which could be ascribed to the low ion conductivity and poor interfaces with electrodes. To further systematically analyze the influence of the water content on the electrochemical performance, the impedances of the Zn | |Zn cells (Supplementary Fig. 21) were measured. For the ZIG-10 wt% based cell, the interface impedance is large. The relatively rigid solid‒solid interface contact caused by the mechanical stiffness of electrolytes generates a large interface impedance. After increasing the water content to 20 wt%, the interface impedance is drastically reduced, which can be primarily attributed to the enhanced ionic transportation at the interface and in the electrolyte and the improved interface due to the extensive area contact. When the water content was further increased to 40 wt% and 60 wt%, the interface impedances slightly decreased due to the further enhanced ionic transportation and interface compatibility.

Furthermore, Zn | |Zn symmetric cells were used to investigate the impact of water content on the reversibility and stability of the Zn anode. As shown in Supplementary Fig. 22, the stability of the ZIG-10 wt% based cell exhibits the largest plating overpotential (approximately 2.5 V), which may be induced by the poor kinetics and vast interface impedance. For the ZIG-20 wt% based cell, a stable cycle of approximately 900 h was obtained (Fig. 3b), which is highly competitive with the reported hydrogel electrolytes (Supplementary Table 1). However, after the water content was increased to 40 wt% and 60 wt %, the cycle lifetimes were markedly decreased to 400 h and 20 h, respectively. The large amount of water in ZIG-40 wt% and ZIG-60 wt% leads to unfavorable Zn plating morphologies (Supplementary Fig. 23). The mossy Zn dendrites tend to form dead Zn and even short circuits induced by dendritic penetration. Moreover, the rate performance of ZIG with different water contents was studied with a fixed time of 10 h at current densities of 0.5, 1, 2, 5, and 8 mA cm^−2^ (Fig. 3c). For different current densities, the voltage hysteresis was improved with decreasing water content, which is primarily attributed to the reduced ionic conductivities and increased interface impedance. Notably, ZIG-60 wt% cannot support a current density of 1 mA cm^−2^ due to drastic side reactions and poor mechanical properties. The impact on Zn plating/stripping behaviour with the ZIG-20 wt% electrolyte was investigated by cyclic voltammetry (CV) (Supplementary Fig. 24) and Zn | |copper (Cu) cell. The coulombic efficiency is an effective method to evaluate the reversibility of Zn plating/stripping. As shown in Fig. 3d and Supplementary Fig. 25, the Zn | |Cu cells were tested at current densities of 0.5 mA cm^−2^ and 0.5 mAh cm^−2^. The ZIG-20 wt% based cell shows stable coulombic efficiencies of ~99% for 300 cycles, indicating an excellent Zn plating/stripping reversibility. When the water content was reduced to 10 wt%, a great fluctuation of coulombic efficiency was achieved due to the large plating overpotential. When the water content was increased to 40 wt%, although ~98% coulombic efficiencies were maintained for ~100 cycles, significant fluctuations were observed in the subsequent cycles, showing poor reversibility. After further the water content was increased to 60 wt%, inferior coulombic efficiencies and limited cycles were achieved. Similarly, the PAA and PAM hydrogels displayed low coulombic efficiencies and failed shortly after a few cycles. Therefore, with increasing water content, the ionic conductivity and interface compatibility can be effectively reinforced, similar to that of HPEs, while the interfacial side reactions, poor reversibility, and undesirable morphologies caused by water become significant barriers to obtaining a stable Zn anode. Additionally, hydrogels with excessively low water content possess properties close to those of SPEs, which leads to poor interface contacts. The lean-water hydrogel offers a balance between HPEs and SPEs, retaining their respective advantages.

**Fig. 4:**
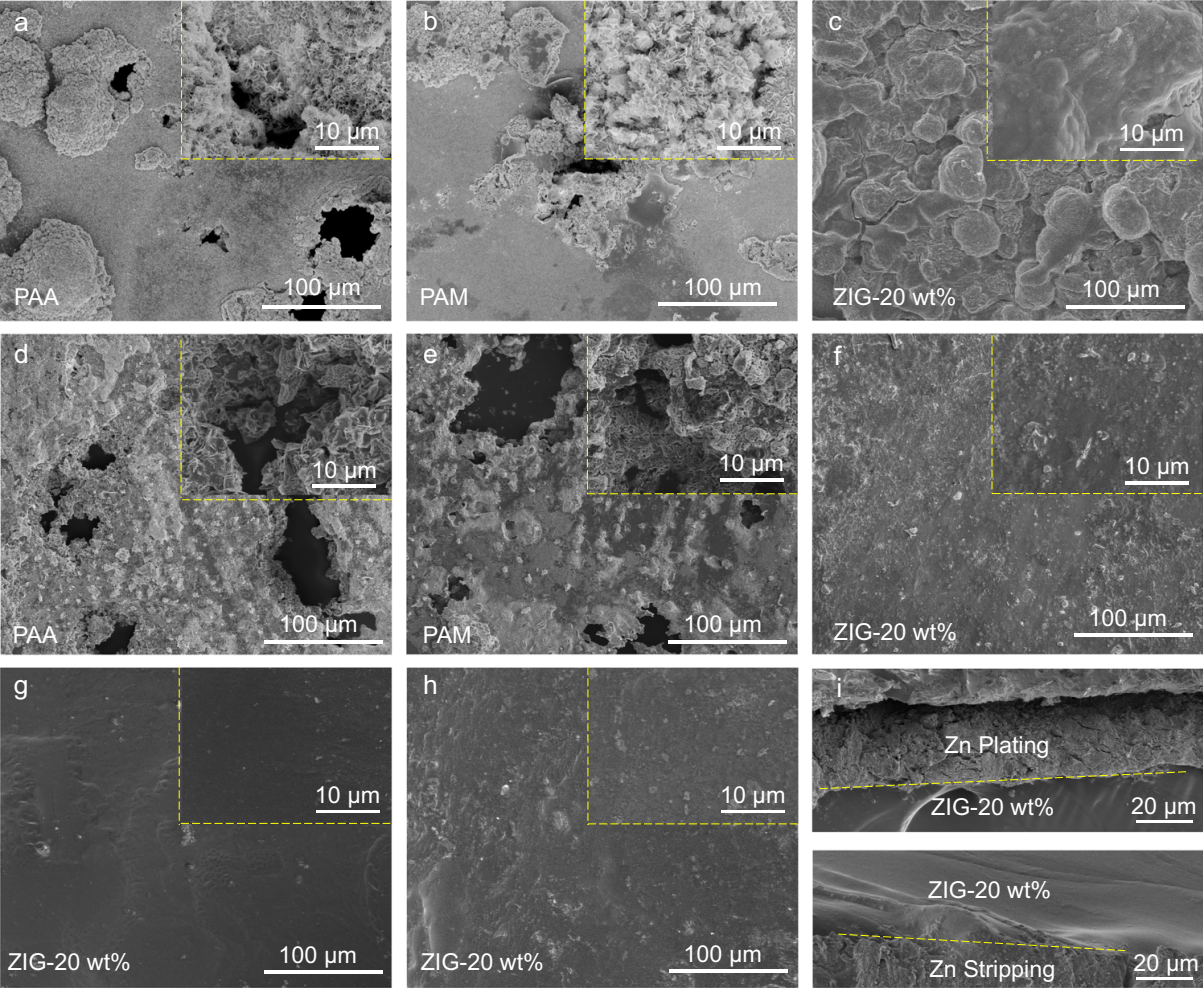
Morphologies of Zn anodes of Zn | |Zn symmetric cells with different electrolytes. **a–c** Top-view SEM images showing the morphology of Zn plating after cycling with 50% Zn utilization. **d–f** Top-view SEM images showing the morphology of Zn stripping after cycling with 50% Zn utilization. Top-view SEM images of ZIG/Zn interfaces after (**g**) Zn plating and (**h**) Zn stripping. **i** Cross-section SEM images of ZIG/Zn interfaces.

Furthermore, a high areal capacity of 3 mAh cm^−2^ was used to evaluate the long-term reversibility of Zn plating/stripping in Zn | |Cu cells. More specifically, two current densities, 1 mA cm^−2^ and 3 mA cm^−2^, were adopted with 6 h and 2 h durations per cycle. As shown in Fig. 3e, relatively long cycle lifetimes (300 cycles and 110 cycles at high and low rates, respectively) and high coulombic efficiencies (~99%) could be achieved based on the ZIG-20 wt% electrolyte, which is highly competitive with the reported hydrogel electrolytes (Supplementary Table 2). In addition, a Zn anode with an areal capacity of ~17 mAh cm^−2^ was used, and the Zn utilization was set to 50% during cycling to further investigate the morphology of the Zn anode by scanning electron microscopy (SEM). For the Zn plated side (Fig. 4a, b), unfavorable and uneven Zn morphologies with distinct nanoflower-like deposits and clearly permeable voids could be observed with PAA and PAM as electrolytes. In contrast, for the ZIG-20 wt% electrolyte-based cell (Fig. 4c), compact and smooth Zn deposition with orientation parallel to the Zn electrode surface forms, indicating a dendrite-suppressed plating mechanism. The Zn morphologies on the Zn-stripped side (Fig. 4d–f) exhibit similar characteristics to those on the plated side. Furthermore, top-view SEM images of the ZIG electrolyte after Zn plating (Fig. 4g) and stripping (Fig. 4h) were investigated to observe the interface between ZIG and Zn. Both retain smooth surfaces without dendrite formations. From the side view SEM images (Fig. 4i), no apparent voids and intimate interface contact between Zn and the ZIG electrolyte can be observed on both the Zn plating and stripping sides, exhibiting integral, dense, and dendrite-free morphologies. In short, benefiting from the lean-water design, the ZIG-20 wt% well supports Zn stripping/plating at various current densities. Furthermore, the side reactions were effectively suppressed, as revealed by the high coulombic efficiency achieved.

### Electrochemical energy storage performance based on the lean-water hydrogel electrolyte

**Fig. 5:**
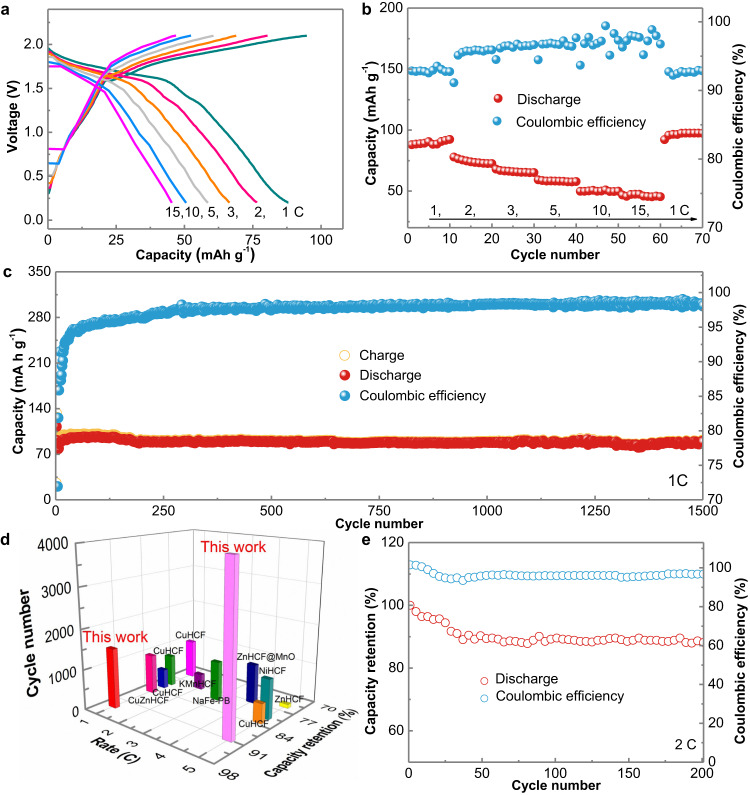
Electrochemical performance of Zn | |MnHCF cell with the ZIG−20 wt% electrolyte. **a** Discharging-charging profiles at different rates. **b** Rate performance and corresponding coulombic efficiency. **c** Cycling performance and coulombic efficiency at 1 C. **d** Comparison of cycling stability among the MnHCF cathode in this work and PBA cathodes reported in aqueous batteries. **e** Cycling performance of a Zn | |MnHCF pouch cell at 2 C.

CV was used to investigate the kinetics of electrochemical performance based on a Zn | |MnHCF full cell, as shown in Supplementary Fig. 26. Two obvious cathodic peaks and two anodic peaks at 1.58 V/1.66 V and 1.9 V/1.98 V were presented, which were ascribed to Mn(III) to Mn(II) and Fe(III) to Fe(II), respectively^36^. Additionally, the relationship between the peak currents and scan rates was inspected, and the slope values of the four peaks were 0.90, 0.86, 0.60 and 0.78, implying that it was controlled by both diffusion and pseudocapacitive behaviours^37^. Furthermore, the galvanostatic discharge/charge (GDC) profiles in Fig. 5a show a voltage plateau at approximately 1.6 V and sloping profiles, confirming the mix of battery− and capacitance-type capacities. The full cell delivered superior rate capabilities of 48-92 mAh g^-1^ at current densities ranging from rates of 15 C to 1 C, which was attributed to the well-retained ionic conductivity of ZIG-20 wt% (Fig. 5b). In addition, as shown in Fig. 5c and Supplementary Fig. 27, the Zn | |MnHCF battery exhibited high coulombic efficiencies of approximately 100% and high-capacity retentions of 91% and 94% after cycling up to 4000 times and 1500 times at rates of 5 C and 1 C, respectively. Notably, the cycling stability and coulombic efficiencies were measured at low current densities, which are more reliable^6^. In contrast, the PAM hydrogel exhibited inferior cycling performance and a low coulombic efficiency (Supplementary Fig. 28). The excellent cycling performance was attributed to the high stability and dendrite-free feature of the Zn anode and the suppressed dissolution of the cathode material with the lean-water design. The ultralong lifespan and high-capacity retentions at 1 C and 5 C significantly outperformed most Prussian blue analogue (PBA)-based batteries with conventional aqueous electrolytes (Fig. 5d)^38–47^. Furthermore, a pouch cell was fabricated to confirm the suppression of side effects and cycled at a rate of 2 C. As shown in Fig. 5e, the battery exhibited negligible capacity decay and approximately 100% coulombic efficiency after 200 cycles. The optical images before and after cycling confirmed that no gas was produced during the charge/discharge processes (Supplementary Fig. 29).

### Adhesion characterizations of the lean-water hydrogel electrolyte

**Fig. 6:**
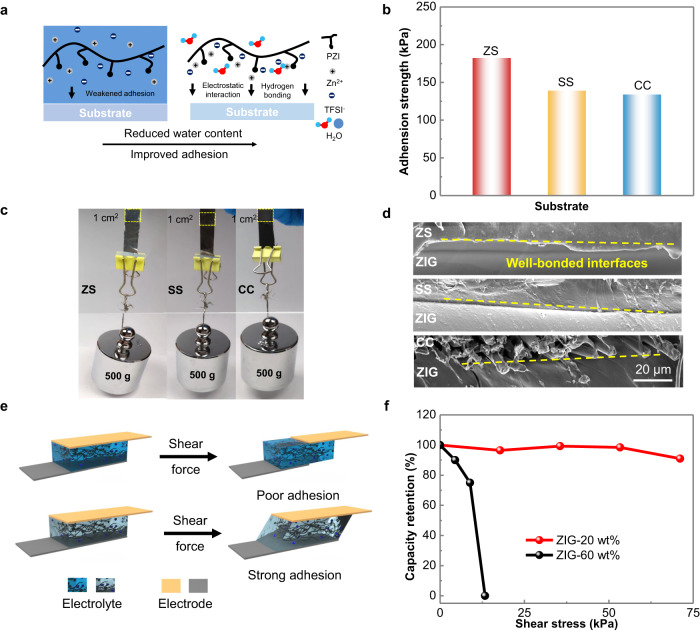
Characterization of the adhesion. **a** Schematic illustration of the adhesive mechanism of ZIG at high and low water contents. **b** Adhesion strength of ZIG−20 wt% with different substrates, Zn sheet (ZS), stainless steel sheet (SS) and carbon cloth (CC). **c** Optical images of ZIG-20 wt% with different substrates bearing a weight of 500 g. **d** SEM images after 5 N shear force on different substrates. **e** Illustration of batteries under shear force. **f** Capacitance retentions of the full battery based on ZIG with 20 wt% and 60 wt% water contents at different shear stresses.

Interestingly, ZIG exhibited excellent adhesion to many material surfaces at a lean-water content. As shown in Fig. 6a, when much free water was present in the polymer matrix, the polar zwitterion groups and Zn salts (adhesion sites) were completely immersed in a large amount of water molecules, resulting in weakened interface adhesion. In contrast, the lean-water content could achieve strong interface adhesion due to the formation of long ion-chain complexes between polar zwitterion groups and Zn salts, which could be attributed to the possible van der Waals forces, hydrogen bonding, electrostatic interactions, and ion-polar interactions at the interfaces^48,49^. The lap-shear method was used to evaluate the adhesion strength of ZIG-20 wt% with the substrate/hydrogel/substrate structure (Supplementary Fig. 30). The adhesion strength reached 100–200 kPa on the Zn sheet (ZS), stainless steel sheet (SS) and carbon cloth (CC) (Fig. 6b, Supplementary Fig. 31). With a contact area of 1 cm^2^, ZIG-20 wt% could support a weight of 500 g (Fig. 6c). In addition, a 1 cm^2^ interface of ZIG-20 wt% with ZS, SS and CC was subjected to a 5 N shear force for two seconds, and the SEM observation further confirmed sufficient physical contact and well-bonded interfaces (Fig. 6d). In contrast, the PAM and PAA hydrogels exhibited poor adhesive abilities (Supplementary Fig. 32)^50^.

For most flexible devices, the bending test is insufficient for evaluating the interface between electrodes and electrolytes. The shear force is generated at the interfaces of different components of a flexible device when the device is applied with almost any deformations. The reliability of a flexible device under shear force has rarely been investigated. Here, a shear force measurement was conducted for flexible ZIBs with ZIG as an electrolyte. The process is illustrated in Fig. 6e. For the electrolyte with poor adhesion to electrodes, the anode and cathode may be easily separated. On the other hand, a strongly bonded interface may equip the flexible device with tolerance to shear loading. Subsequently, we assessed a Zn | |MnHCF flexible battery based on ZIG with 20 wt% and 60 wt% water contents, and the dependence of capacity on the shear force was measured. The discharge curves in Supplementary Fig. 33 and the capacity retention curve in Fig. 6f reveal a linear decrease in capacity for the device with the ZIG-60 wt% electrolyte with increasing shear stress. After reaching 13 kPa, no capacity remained due to the complete separation of the electrolyte and electrodes. However, for the device based on ZIG-20 wt%, only a small fluctuation was obtained before the shear stress reached 71 kPa (Fig. 6f), and then the capacity retention slightly decreased to 90% due to tiny sliding. This result shows the excellent adhesive ability of ZIG under lean-water conditions, which is promising for maintaining clingy interfaces in flexible/wearable devices^51^.

## Discussion

In summary, HPEs and SPEs have been widely explored for solid-state/quasi-solid-state ZIBs. However, much water is inevitably incorporated for HPEs to achieve rapid ionic transportation; thus, the HPEs only alleviate rather than eliminate the side reactions of the Zn anode. On the other hand, SPEs suffer from low ionic conductivities and high interface impedances, although they generally provide good stability. Herein, we provide an important balance between the two by developing a lean-water hydrogel electrolyte, which sits between conventional HPEs and SPEs. ZIG is prepared with covalently bonded positively and negatively charged groups as the polymer skeleton and zinc salt as the coordination unit. A special ion channel can be constructed. After introducing a small amount of water, significantly improved dissociation and transportation of Zn^2+^ can be achieved through a lubrication mechanism, resulting in an impressive ionic conductivity (2.6 × 10^−3^ S cm^−1^) even under lean-water conditions. The manipulation of the O-H structure in water molecules prominently expands the electrochemical stability window and suppresses side reactions at the anode side. Additionally, highly reversible Zn plating/stripping demonstrates the excellent stability of the anode. Owing to these merits, the full cell obtains excellent cycling stability of 4000 and 1500 cycles and superior capacity retention of 91% and 94% at rates of 5 C and 1 C, respectively. Moreover, the excellent adhesive ability of the hydrogel electrolyte to electrodes under lean-water conditions can equip flexible batteries with excellent tolerance to shear stress. Our results offer a type of quasi-solid-state electrolyte, which provides an ionic conductivity close to that of a conventional hydrogel, as well as wide electrochemical stability windows and superior suppression to side reactions at the Zn anode close to those of SPEs. The important balance point set by the lean-water hydrogel may equip aqueous electrolyte batteries with superior rateability and high cycling stability.

## Methods

### Materials

1-Vinylimidazole (≥97%), 1,3-Propanesultone (≥99%), poly(ethyleneglycol) dimethacrylate (PEGDMA, Mn = 550), 2-Hydroxy-4’-(2-hydroxyethoxy)-2-methylpropiophenone (99%), Acetonitrile (99%) were purchased from Aladdin, Zinc di[bis(trifluoromethylsulfonyl)imide] (98%) was obtained from Energy Chemical. The materials in the experiment were used without further purification.

### Synthesis of VIPS and ZIG

The 3-(1-vinyl-3-imidazolio) propanesulfonate (VIPS) zwitterionic monomer was synthesized as the method below. Under the protection of argon, the 1-vinylimidazole (0.2 mol) was added in dry acetonitrile solution, and then 1,3-propanesultone (0.2 mol) was dropwise in the solution at 0 °C. After stirring for about 3 days, the product was filtered and washed with acetonitrile. The purified product was finally dried under vacuum at room temperature. The ZIG was prepared via a UV-assisted copolymerization. The homogeneous solutions were prepared by mixing VIPS and Zn(TFSI)_2_ in a mole ratio of 2:1, PEGDMA (5 wt% to the monomers) as the cross-linker, and 2-hydroxy-4’-(2-hydroxyethoxy)-2-methylpropiophenone (0.5 wt% to the monomers) as the photo-initiator in an aqueous solution. The mixture solution was injected into a home-made quartz mold, sealed, and irradiated by a UV lamp for 30 min at room temperature to produce free-standing polymeric films. For the determination of water content, the film was dried in a vacuum oven at 100 °C for 24 h to achieve the weight of m_0_, and then adding different water weights to achieve the weight of m_t_, the final water content is equal to (m_t_-m_0_) /m_t_ × 100%. The PAA and PAM hydrogels used in our work possess ~80 wt% and ~90 wt% water contents, respectively.

### Synthesis of manganese hexacyanoferrate (MnHCF)

MnHCF particles were prepared according to the method below. MnSO_4_ (0.12 M) (≥99.99%, Aladdin) was dissolved into 200 ml aqueous solution, and then was added dropwise into 200 ml of K_3_Fe(CN)_6_ (0.06 M) (≥99.95%, Aladdin) aqueous solution. Next, the mixture was stirred at 60 °C for 30 min. After standing for about 1 h, the precipitates could be achieved by centrifugation and purified with water and ethanol. After further drying under a vacuum at 60 °C, the brown solids could be achieved. Typically, the cathode was prepared by coating the slurry by mixing MnHCF powers, super p (conductive agent) and polyvinylidene fluoride (PVDF, >99.5%, HSV900, Arkema) (7:2:1 wt/wt/wt) on a piece of carbon cloth. Then, the electrodes were dried in a vacuum oven at 60 °C for 12 h. The mass loading of MnHCF is around 1–2 mg cm^−2^.

### Characterization methods

Field-emission scanning electron microscope (FESEM; Verios 5UC) was performed to observe the surface and cross-section of the sample. Thermogravimetric Analysis (TGA) was applied to investigate the thermal properties. The side-effect of decomposition on the Zn surface was confirmed by X-ray diffraction (XRD, Bruker D2 Phaser). Differential scanning calorimetry (DSC) (Q2000 TA Instruments) was conducted to obtain the phase transition behaviors. Bruker 600 MHz was conducted to obtain ^67^Zn nuclear magnetic resonance (NMR) spectra. Fourier-transform infrared spectroscopy (FTIR, INVENIO-R) was performed to analyze the different characteristics. The gas pressure experiments were performed on a gas pressure sensor which was connected with a sealed electrochemical cell.

### Electrochemical measurements

All electrochemical measurements were conducted at an average temperature of 28 °C ± 1 °C. The electrochemical tests were carried out in a climatic/environmental chamber. The ionic conductivity was tested by placing the electrolyte in the middle of two stainless steels (SS) and it is equal to L/(A×R) where L is the thickness and A is the surface area of the membrane.

Linear sweep voltammetry (LSV) measurements were carried out by a CHI 760D electrochemical workstation. The Zn metal and Ti were severed as reference and the working electrodes, respectively, and the tests were performed from the original potential to 3.0 V and the original potential to −0.5 V at a scan rate of 0.5 mV s^−1^.

The coin-2032 cells used a zinc foil (50 µm, ≥99.99%, Yudingda Metal, Φ10 mm) as the anode electrode, MnHCF on carbon clothes (Φ10 mm) as the cathode, and the ZIG (~300 µm, Φ14 mm) as the electrolyte. The areal current density of ~0.16 mA cm^−2^ cycled for the full battery corresponds to 1 C. The cutoff voltages were set as 0.2 V and 2.1 V, separately. For the pouch cell, zinc foil (4 cm × 4 cm × 100 µm) was used as the anode, MnHCF on carbon clothe (4 cm × 4 cm) as the cathode. The galvanostatic cycling was characterized by a LAND CT2001A Battery Testing System.

In-situ optical microscope studies were conducted to visualize gas evolution behavior on Zn electrode with PAM, PAA and ZIG- 20 wt% electrolytes. Two Zn electrodes were stuck on a glass slide, and they were separated by different electrolytes with a length of ~2 mm. Another slide was then put on the glass slide and the device was placed under a microscope equipped with a digital camera. A current density of 5 mA cm^−2^ was set through an electrochemical workstation.

### Adhesion test

The experiment was conducted by a universal machine (UTM2202) with a standard lap-shear method. The ZIG-20 wt% electrolyte was cut into a required square (1–2 cm^2^) and placed in the middle of two substrates. After giving gentle pressure (~10 kPa), the specimens were tested with a speed of 10 mm min^−1^. The maximum force could be read in the curves and the adhesion strength could be achieved by dividing the maximum force by the adhesion area.

### Shear force measurement

Two unpacked batteries with ZIG-20 wt% and ZIG-60 wt% as electrolytes, MnHCF on carbon cloth (1.5 cm × 1.5 cm) as cathode and Zn foil (1.5 cm × 1.5 cm × 100 µm) as anode were put on a tensimeter, and then the cathode and anode sides were positioned on the opposite clinchers, separately. The shear force can be observed in the tensimeter after changing the distance. After gradually increasing the force to different values, the corresponding discharge capacity can be obtained by LAND Battery Testing System. Finally, the battery went into fracture.

## Supplementary information

Supplementary Information

## Data Availability

The data that support the findings of this study are available within the text including the Methods, and Supplementary information. Raw datasets related to the current work are available from the corresponding author on reasonable request.

## References

[1] Zhang N (2020). Materials chemistry for rechargeable zinc-ion batteries. Chem. Soc. Rev..

[2] Jiang L, Dong D, Lu YC (2022). Design strategies for low temperature aqueous electrolytes. Nano Res. Energy.

[3] Tang X (2021). A universal strategy towards high-energy aqueous multivalent-ion batteries. Nat. Commun..

[4] Yang F (2022). Understanding H2 evolution electrochemistry to minimize solvated water impact on zinc anode performance. Adv. Mater..

[5] Tribbia M, Glenneberg J, Zampardi G, La Mantia F (2022). Highly efficient, dendrite‐free zinc electrodeposition in mild aqueous zinc-ion batteries through indium‐based substrates. Batteries Supercaps..

[6] Zampardi G, La Mantia F (2022). Open challenges and good experimental practices in the research field of aqueous Zn-ion batteries. Nat. Commun..

[7] Qiu H (2022). Eutectic crystallization activates solid-state zinc-ion conduction. Angew. Chem. Int. Ed..

[8] Wang X (2021). Advances and perspectives of cathode storage chemistry in aqueous zinc-ion batteries. ACS Nano.

[9] Li H (2019). Advanced rechargeable zinc-based batteries: recent progress and future perspectives. Nano Energy.

[10] Li Q (2022). Dendrite issues for Zn anodes in a flexible cell configuration. Angew. Chem. Int. Ed..

[11] Wang J (2021). Room-temperature fast zinc-ion conduction in molecule-flexible solids. Mater. Today Energy.

[12] Zhao Z (2021). In-situ formed all-amorphous poly (ethylene oxide)-based electrolytes enabling solid-state Zn electrochemistry. Chem. Eng. J..

[13] Lv Y, Xiao Y, Ma L, Zhi C, Chen S (2021). Recent advances in Electrolytes for “Beyond Aqueous” zinc-ion batteries. Adv. Mater..

[14] Zhao S (2021). Multi‐functional hydrogels for flexible zinc-based batteries working under extreme conditions. Adv. Energy Mater..

[15] Liumin S (2015). “Water-in-salt” electrolyte enables high-voltage aqueous lithium-ion chemistries. Science.

[16] Wang Y (2022). Enabling high-energy-density aqueous batteries with hydrogen bond-anchored electrolytes. Matter.

[17] Wang F (2018). Highly reversible zinc metal anode for aqueous batteries. Nat. Mater..

[18] Xie J, Liang Z, Lu YC (2020). Molecular crowding electrolytes for high-voltage aqueous batteries. Nat. Mater..

[19] Hu E YX (2018). Rejuvenating zinc batteries. Nat. Mater..

[20] Mu L (2019). Structural strategies to design bio-ionic liquid: tuning molecular interaction with lignin for enhanced lubrication. J. Mol. Liq..

[21] Silvester DS (2021). Electrical double layer structure in ionic liquids and its importance for supercapacitor, battery, sensing, and lubrication applications. J. Phys. Chem. C..

[22] Hsu SM (2004). Molecular basis of lubrication. Tribology Int..

[23] Ma Q, Qi P, Dong G (2021). An experimental and molecular dynamics study of the superlubricity enabled by hydration lubrication. Appl. Surf. Sci..

[24] Kim H-J, Seo K-J, Kang KH, Kim D-E (2016). Nano-lubrication: a review. Int. J. Precis. Eng. Manuf..

[25] Li G (2020). Polysulfide regulation by the zwitterionic barrier toward durable lithium-sulfur batteries. J. Am. Chem. Soc..

[26] D’Angelo AJ, Panzer MJ (2018). Decoupling the ionic conductivity and elastic modulus of gel electrolytes: fully zwitterionic copolymer scaffolds in lithium salt/ionic liquid solutions. Adv. Energy Mater..

[27] Lind F, Rebollar L, Bengani-Lutz P, Asatekin A, Panzer MJ (2016). Zwitterion-containing ionogel electrolytes. Chem. Mater..

[28] Tiyapiboonchaiya C (2004). The zwitterion effect in high-conductivity polyelectrolyte materials. Nat. Mater..

[29] Mo F (2020). Zwitterionic sulfobetaine hydrogel electrolyte building separated positive/negative ion migration channels for aqueous Zn‐MnO2 batteries with superior rate capabilities. Adv. Energy Mater..

[30] Schlenoff JB (2014). Zwitteration: coating surfaces with zwitterionic functionality to reduce nonspecific adsorption. Langmuir.

[31] Chen S, Li L, Zhao C, Zheng J (2010). Surface hydration: principles and applications toward low-fouling/nonfouling biomaterials. Polymer.

[32] Li ZH (2010). Effect of zwitterionic salt on the electrochemical properties of a solid polymer electrolyte with high temperature stability for lithium ion batteries. Electrochim. Acta.

[33] Park H (2010). Spectroscopic and computational insight into the intermolecular interactions between Zwitter-type ionic liquids and water molecules. Chemphyschem.

[34] Nguyen DQ (2007). Multi-functional zwitterionic compounds as additives for lithium battery electrolytes. Electrochem. Commun..

[35] Dubouis N (2020). Tuning water reduction through controlled nanoconfinement within an organic liquid matrix. Nat. Catal..

[36] Donghong W (2021). A manganese hexacyanoferrate framework with enlarged ion tunnels and two-species redox reaction for aqueous Al-ion batteries. Nano. Energy.

[37] Yang Q (2019). Activating C-coordinated iron of iron hexacyanoferrate for Zn hybrid-ion batteries with 10 000-cycle lifespan and superior rate capability. Adv. Mater..

[38] Lu K, Song B, Zhang Y, Ma H, Zhang J (2017). Encapsulation of zinc hexacyanoferrate nanocubes with manganese oxide nanosheets for high-performance rechargeable zinc ion batteries. J. Mater. Chem. A..

[39] Gupta T (2016). Improving the cycle life of a high-rate, high-potential aqueous dual-ion battery using hyper-dendritic zinc and copper hexacyanoferrate. J. Power Sources.

[40] Wang L-P (2017). Prussian blue nanocubes as cathode materials for aqueous Na-Zn hybrid batteries. J. Power Sources.

[41] Lu K, Song B, Zhang J, Ma H (2016). A rechargeable Na-Zn hybrid aqueous battery fabricated with nickel hexacyanoferrate and nanostructured zinc. J. Power Sources.

[42] Zhang L, Chen L, Zhou X, Liu Z (2015). Towards high-voltage aqueous metal-ion batteries beyond 1.5 V: the zinc/zinc hexacyanoferrate system. Adv. Energy Mater..

[43] Deng W (2021). Zn2+ induced phase transformation of K 2 MnFe(CN) 6 boosts highly stable zinc-ion storage. Adv. Energy Mater..

[44] Kasiri G, Glenneberg J, Bani Hashemi A, Kun R, La Mantia F (2019). Mixed copper-zinc hexacyanoferrates as cathode materials for aqueous zinc-ion batteries. Energy Storage Mater..

[45] Kasiri G, Glenneberg J, Kun R, Zampardi G, La Mantia F (2020). Microstructural changes of prussian blue derivatives during cycling in zinc‐containing electrolytes. ChemElectroChem.

[46] Zampardi G (2021). Effect of the reactants concentration on the synthesis and cycle life of copper hexacyanoferrate for aqueous Zn-ion batteries. Electrochem. Commun..

[47] Trócoli R, Kasiri G, La Mantia F (2018). Phase transformation of copper hexacyanoferrate (KCuFe(CN)6) during zinc insertion: Effect of co-ion intercalation. J. Power Sources.

[48] Chen H (2018). Exploring chemical, mechanical, and electrical functionalities of binders for advanced energy-storage devices. Chem. Rev..

[49] Zou F, Manthiram A (2020). A review of the design of advanced binders for high‐performance batteries. Adv. Energy Mater..

[50] Gu C (2021). Small molecule-based supramolecular-polymer double-network hydrogel electrolytes for ultra-stretchable and waterproof Zn–air batteries working from −50 to 100 °C. Energy Environ. Sci..

[51] Qi Y (2021). Categorizing wearable batteries: Unidirectional and omnidirectional deformable batteries. Matter.

