# Supplementary material for: Lean-water hydrogel electrolyte for zinc ion batteries
Source: Nat Commun. 2023 Jul 1;14:3890. doi: 10.1038/s41467-023-39634-8 (PMC10314915; doi:10.1038/s41467-023-39634-8)
Supplement: Supplementary file 1 — Supplementary Information [file 41467_2023_39634_MOESM1_ESM.pdf]

Supporting Information

Lean-Water Hydrogel Electrolyte for Zinc Ion Batteries

Yanbo Wang[1], Qing Li[1], Hu Hong[1], Shuo Yang[1], Rong Zhang[1], Xiaoqi Wang[2], Xu Jin[*2], Bo Xiong[2], Shengchi Bai[2] and Chunyi Zhi [*1,3,4]

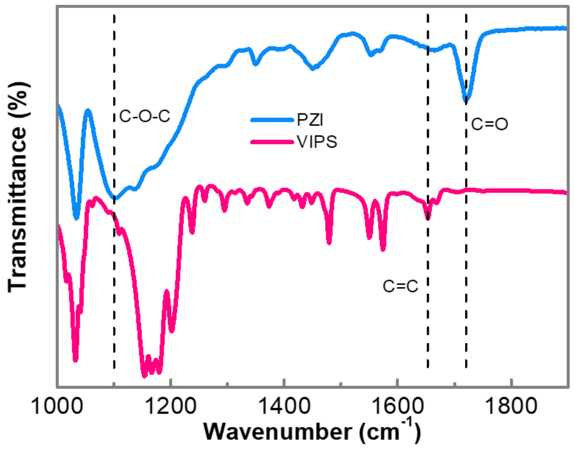

Supplementary Figure 1. FTIR spectra of the VIPS and PZI. The characteristic peak at 1650 cm^{-1} was nearly disappeared after polymerization, proving the open of C=C bond. Two new peaks of 1101 and 1722 cm^{-1} are ascribed to C-O-C and C=O vibrations, respectively, implying the occurrence of polymerization.

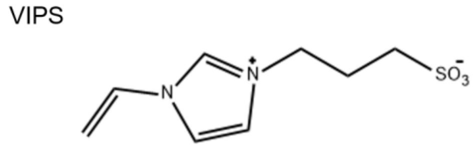

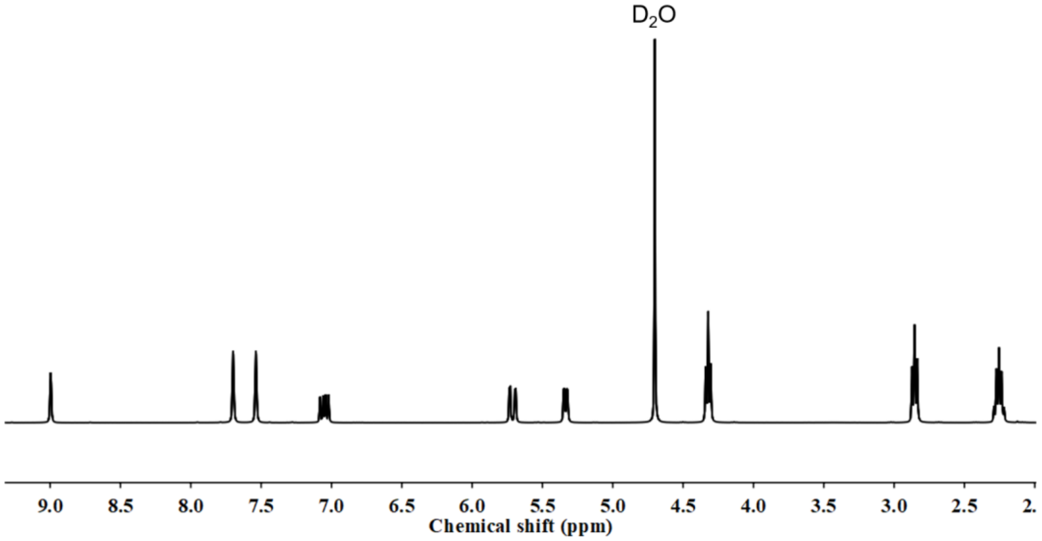

Supplementary Figure 2. ^{1}H-NMR spectra of VIPS in D_2O. The observed resonance peaks were consistent with the expected structure, indicating a successful reaction.

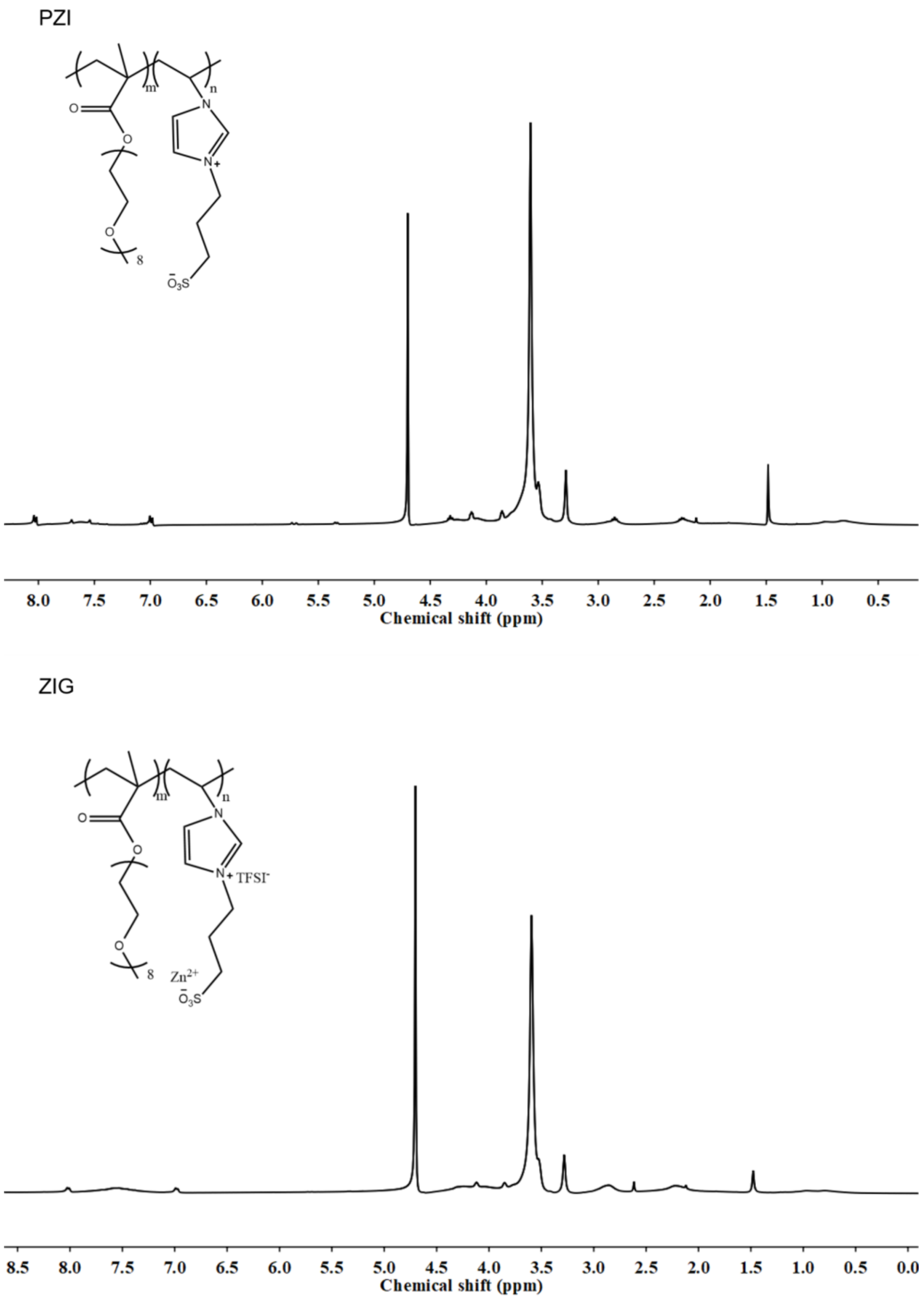

Supplementary Figure 3. ^{1}H-NMR spectra of PZI and ZIG in D_2O. The characteristic resonances of vinyl groups, which appear at δ = 5.75 ppm and 5.35 ppm, were nearly absent after polymerization, indicating a successful polymer reaction.

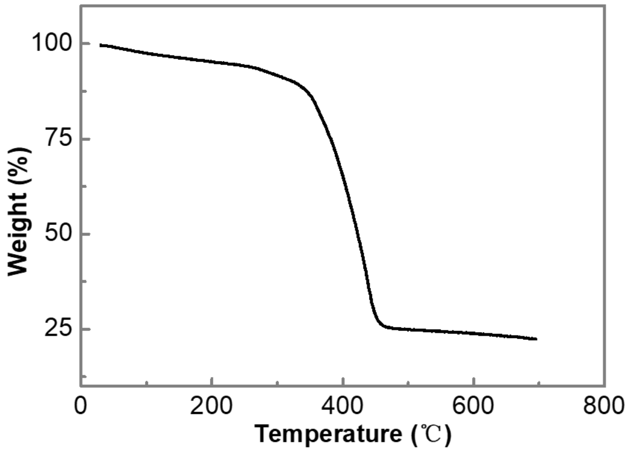

Supplementary Figure 4. TGA plot of ZIG.

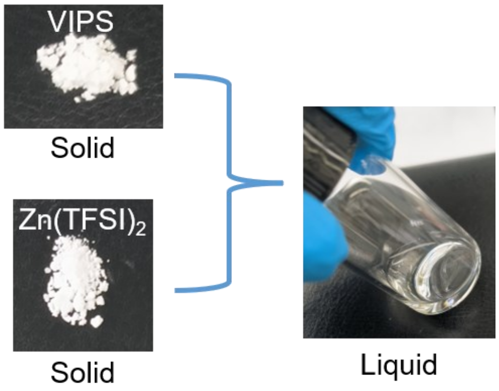

Supplementary Figure 5. Photographs of imidazolium-based zwitterion VIPS and zinc salt. After mixing VIPS and salt in water solution and then evaporating water, a change from solid-state to liquid-state can be obtained.

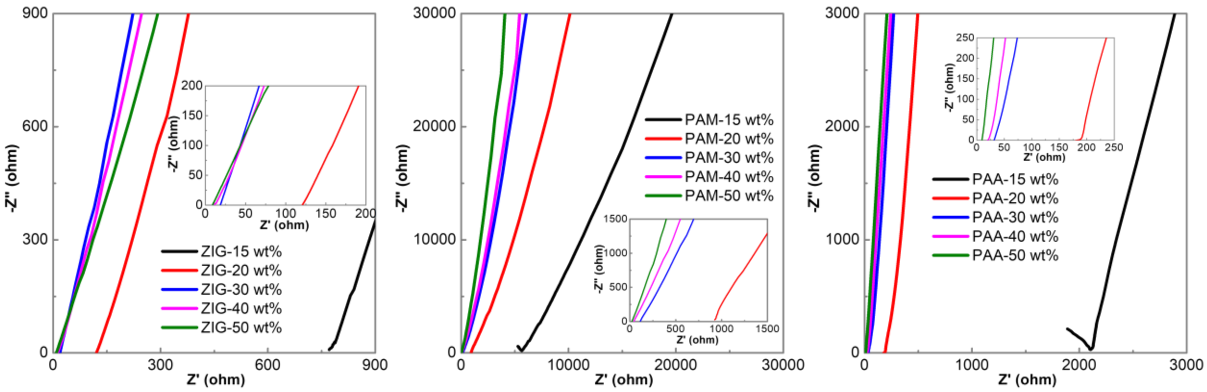

Supplementary Figure 6. Electrochemical impedance spectra. Electrochemical impedance spectra of the ZIG, PAM and PAA with different water.

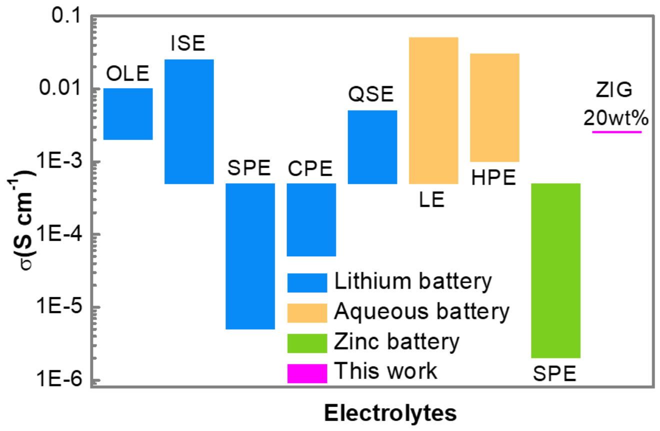

Supplementary Figure 7. The comparison of ionic conductivities of different electrolytes.[1-13] The OLE, ISE, SPE, CPE, QSE, LE, and HPE represent organic liquid electrolytes (lithium ion batteries), inorganic solid electrolytes (lithium ion batteries), solid polymer electrolytes (blue bar for lithium ion batteries, green bar for zinc based batteries), composite polymer electrolytes (lithium ion batteries), quasi-solid-state electrolytes (lithium ion batteries), liquid electrolytes (aqueous batteries), and hydrogel electrolytes (aqueous batteries), respectively.

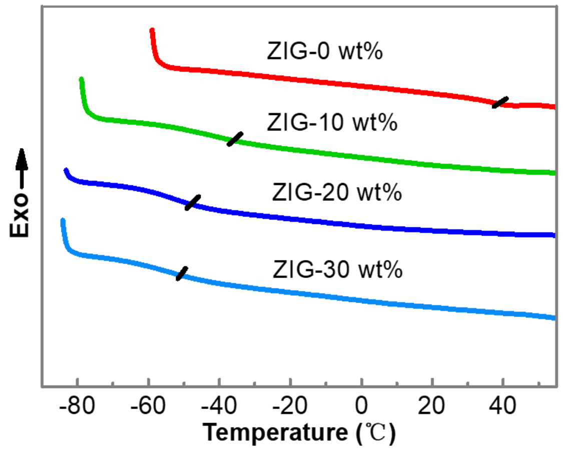

Supplementary Figure 8. DSC test of ZIG with different water contents at 10℃ min^{-1}.

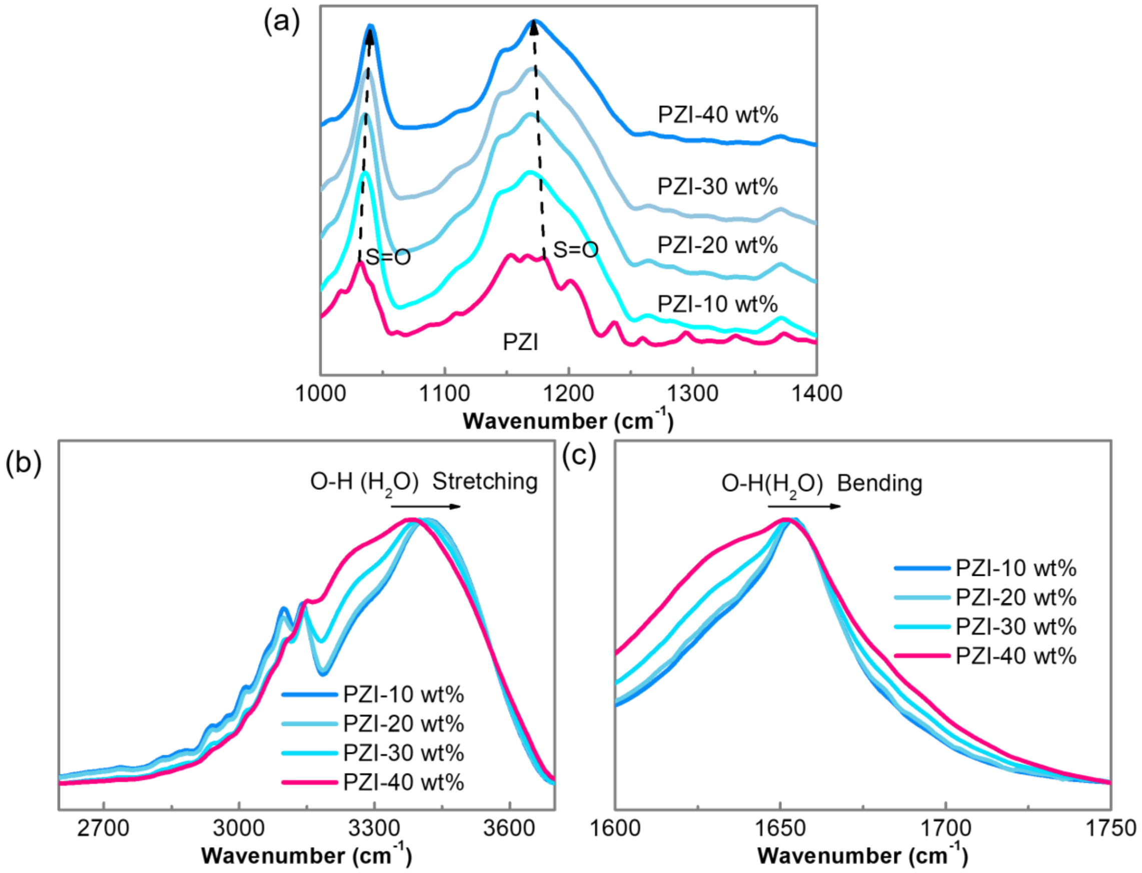

Supplementary Figure 9. FTIR analysis. a FTIR spectra of PZI without and with different water contents. **b** the stretching mode of normalized FTIR spectra of PZI with different water contents. **c** the bending mode of normalized FTIR spectra of PZI with different water contents.

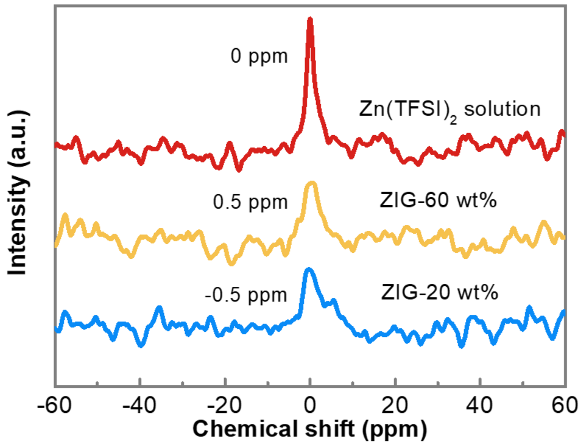

Supplementary Figure 10. ^{67}Zn nuclear magnetic resonance (NMR) spectra of the ZIG electrolytes with different water contents.

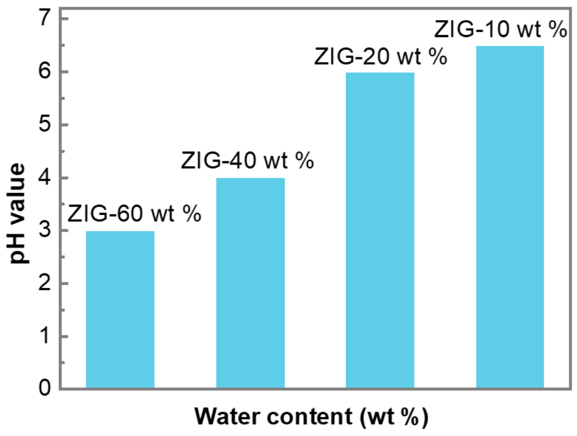

Supplementary Figure 11. pH values of the ZIG electrolytes with different water contents.

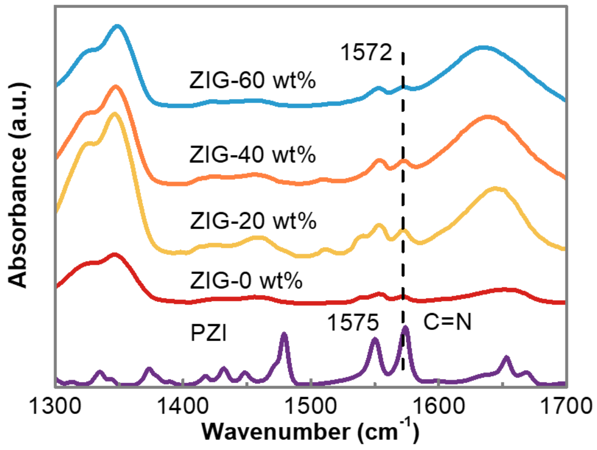

Supplementary Figure 12. FTIR spectra of PZI and ZIG with different water contents. The peak at 1575 cm^{-1} for PZI is assigned to the C=N in imidazole.

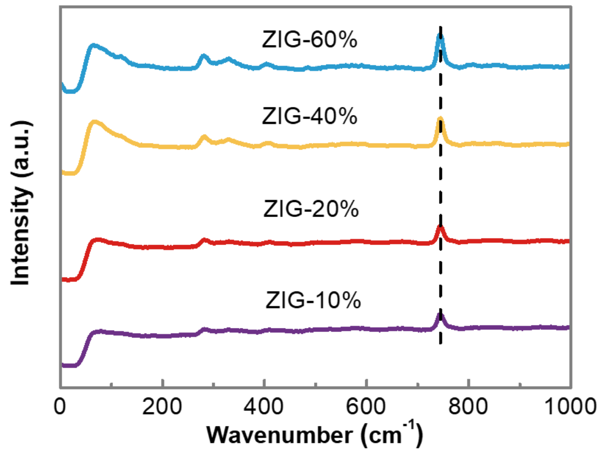

Supplementary Figure 13. Raman spectra of ZIGs with different water contents. The Raman vibration at ~745 cm^{-1} is assigned to the S-N-S bending vibration in $TFSI^-$.

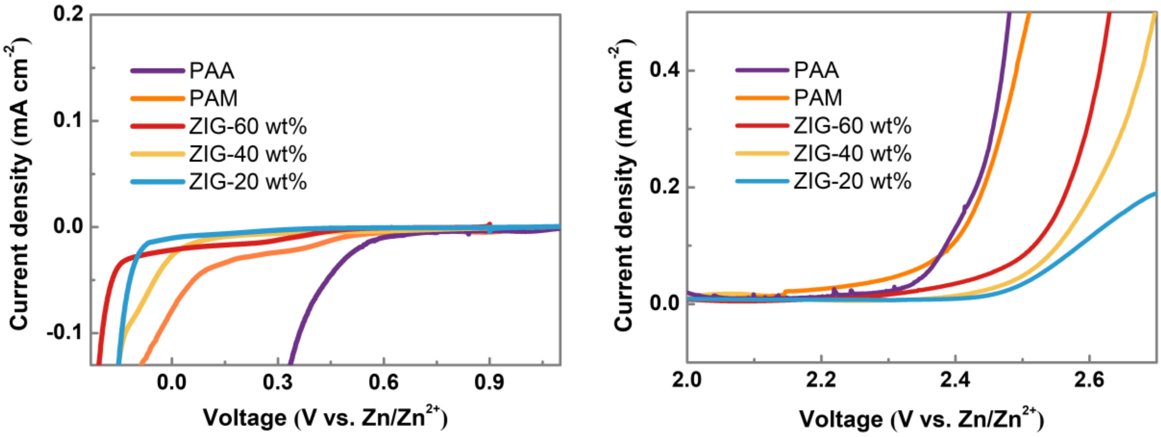

Supplementary Figure 14. The corresponding magnified view of linear sweep voltammetry near anodic and cathodic extremes.

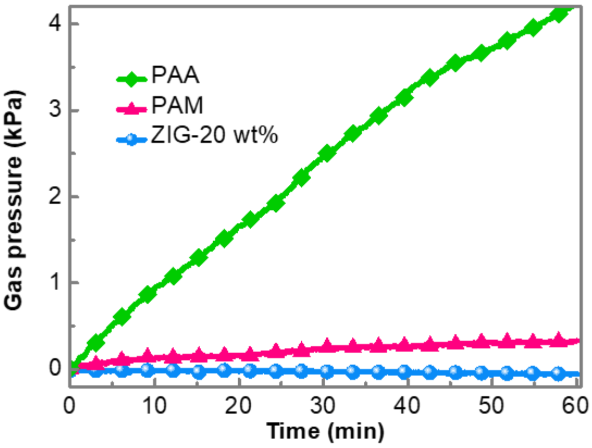

Supplementary Figure 15. The gas evolution for the ZIG-20 wt%, PAM and PAA (conventional HPEs).

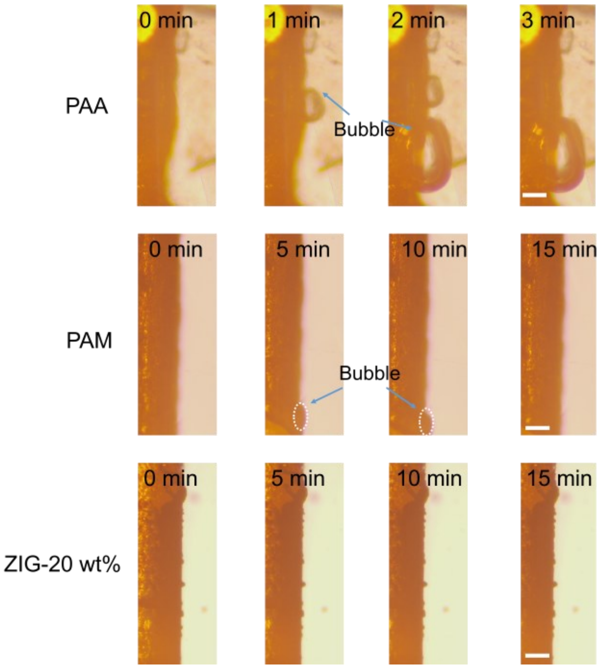

Supplementary Figure 16. In situ optical microscope images of the Zn anode with different electrolytes, scale bar: 50 μm.

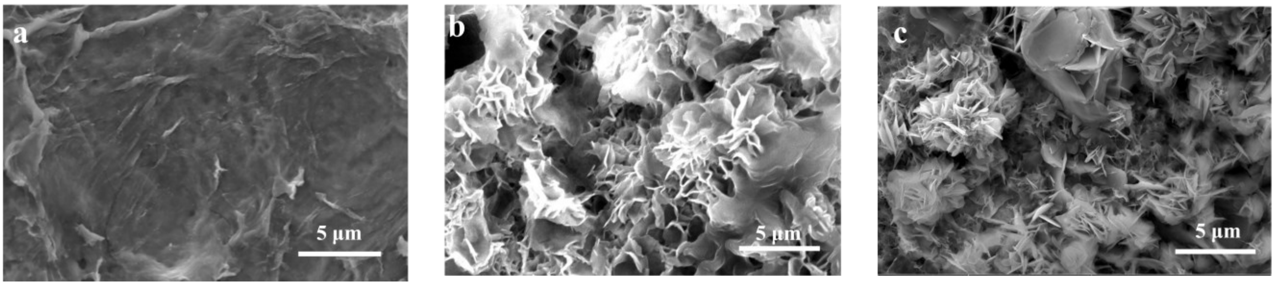

Supplementary Figure 17. SEM images of Zn anode after plating/striping for **a** ZIG-20 wt% **b** PAM hydrogel and **c** PAA hydrogel.

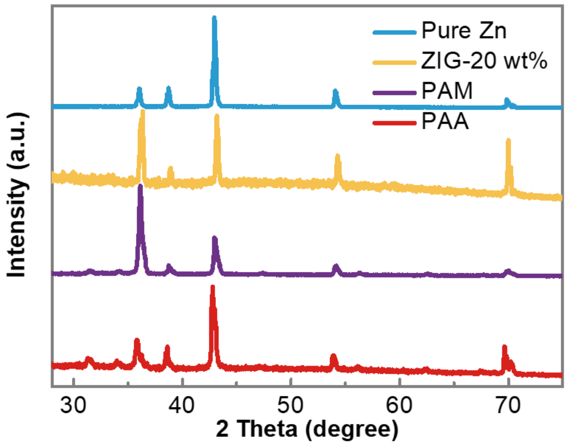

Supplementary Figure 18. XRD pattern of Zn anode after plating/striping for ZIG-20 wt%, PAM hydrogel and PAA hydrogel.

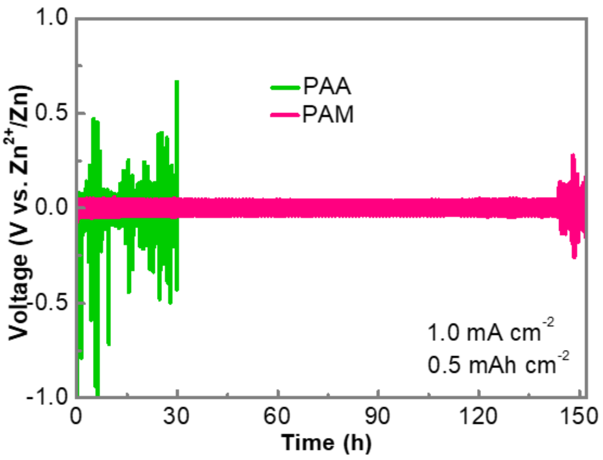

Supplementary Figure 19. The galvanostatic Zn plating/stripping in Zn||Zn symmetrical cells with PAM and PAA electrolytes (conventional HPEs). The limited cycles correspond to the gas evolution. The bubble can block the surface of the electrode and hinder the nucleation of Zn, resulting in increased overpotentials, uneven Zn deposition, and growth of dendrite.

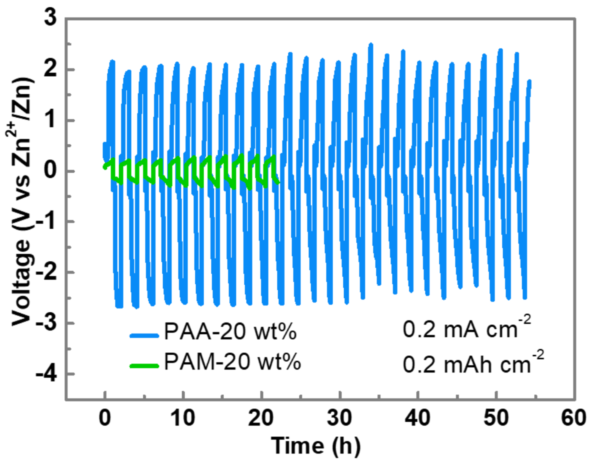

Supplementary Figure 20. Comparison of voltage-time profiles of Zn||Zn symmetric cells based on PAA and PAM with 20 wt% water content.

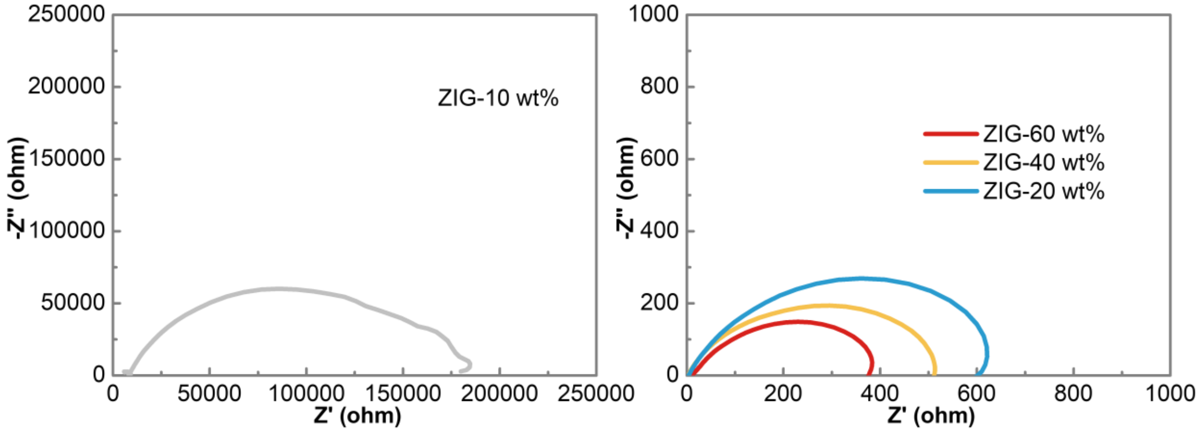

Supplementary Figure 21. EIS results of the Zn||Zn symmetric cells based on the ZIGs with different water contents.

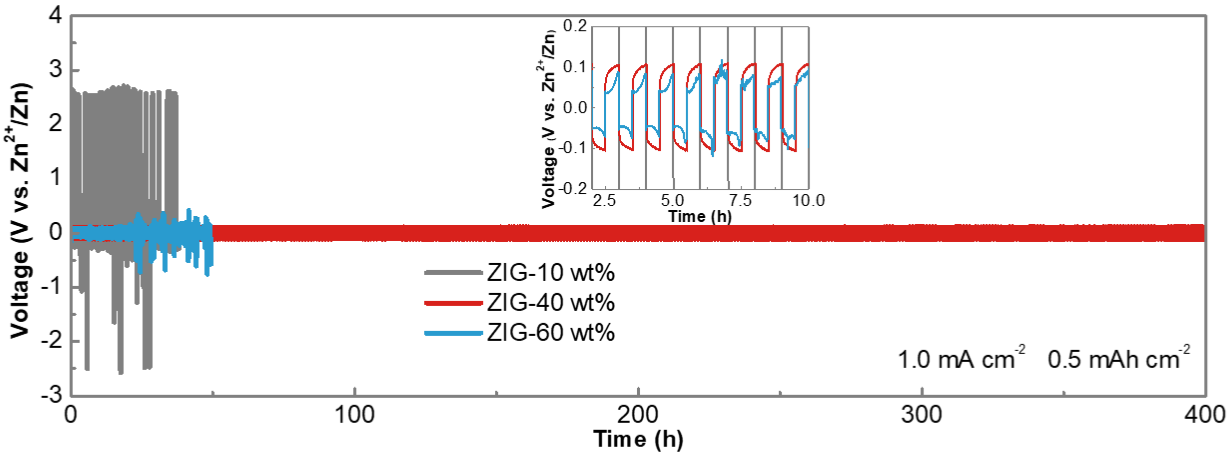

Supplementary Figure 22. Galvanostatic cycling of Zn||Zn symmetrical cells at 1 mA cm^{-2} in the ZIG hydrogel electrolytes with different water contents. The inset displays the enlarged voltage profiles of the initial cycles.

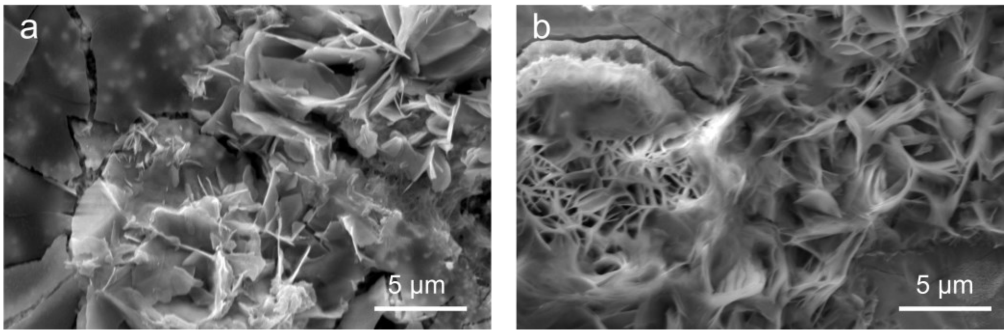

Supplementary Figure 23. SEM images of Zn anode after Zn plating/striping with **a** ZIG-40 wt% and **b** ZIG-60 wt% electrolytes.

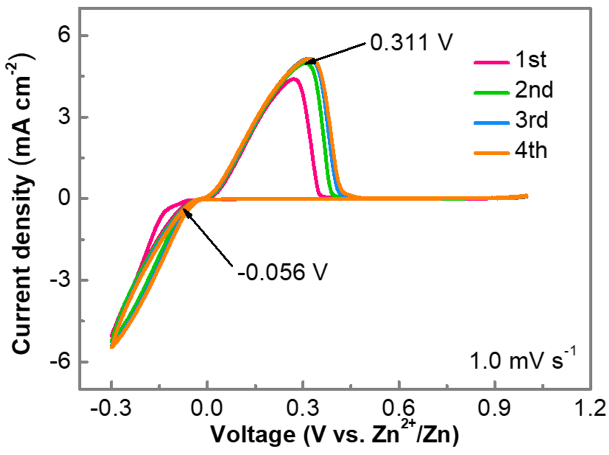

Supplementary Figure 24. CV curves of Zn plating/stripping using a Zn||Cu coin cell based on the ZIG-20 wt%.

Zn is deposited at -0.056 V (vs. Zn^{2+}/Zn) with a low nucleation overpotential, implying a low barrier for Zn deposition and fast kinetic although under a lean-water state. Additionally, it can be noted that the Zn oxidation is almost finished at 0.311 V (vs. Zn^{2+}/Zn).

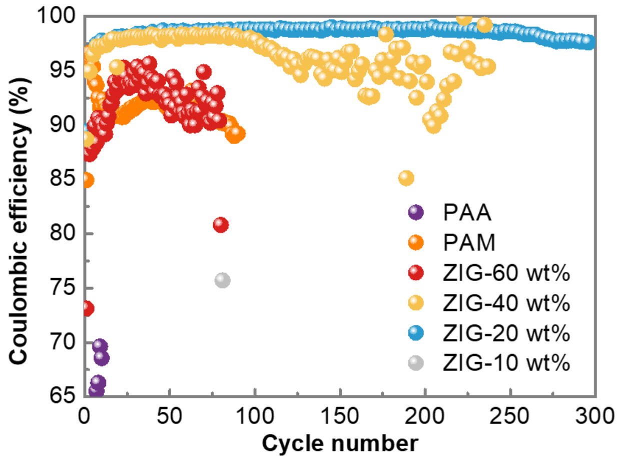

Supplementary Figure 25. The enlarged profile of coulombic efficiencies of Zn deposition in a Zn||Cu cell.

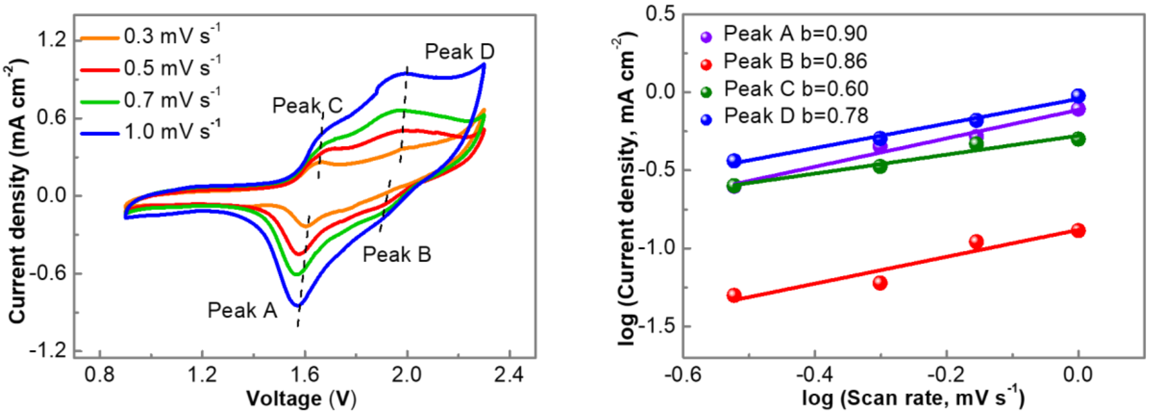

Supplementary Figure 26. CV curves of Zn||MnHCF cell with ZIG-20 wt% electrolyte under different scan rates and the linear fit between log (current, i) and log (scan rate, v) at cathodic and anodic peaks.

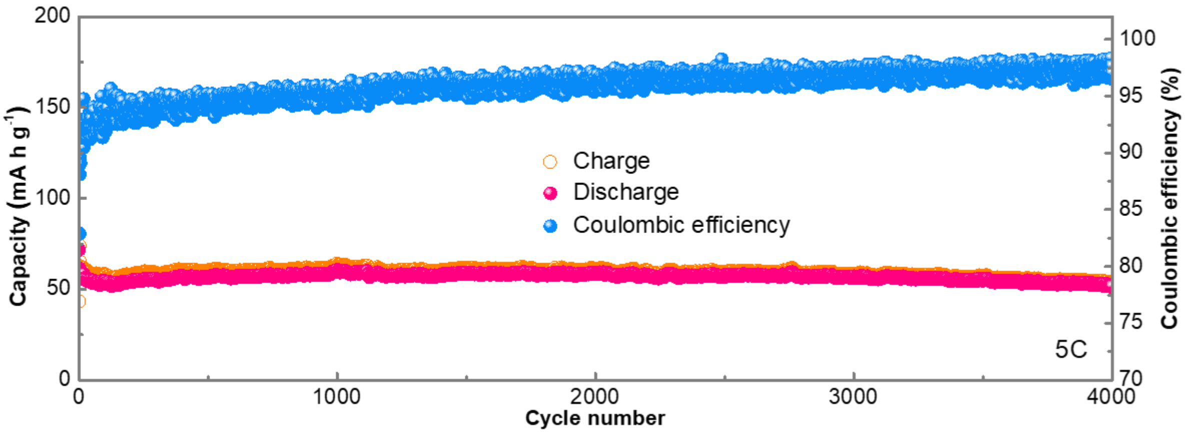

Supplementary Figure 27. Cycling performance and coulombic efficiencies of a Zn||MnHCF cell based on ZIG-20 wt% electrolyte at 5 C.

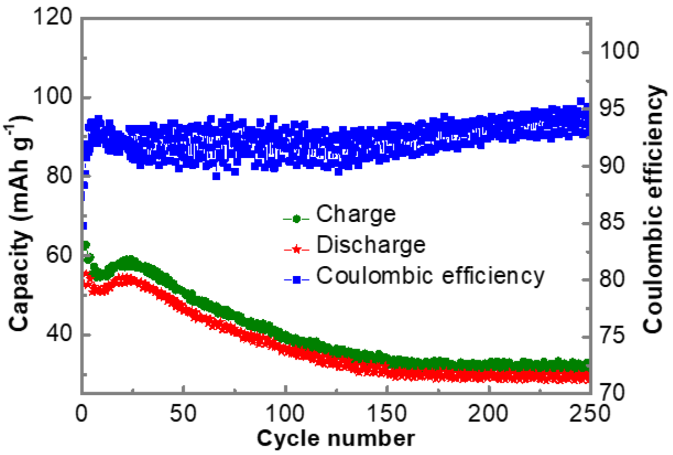

Supplementary Figure 28. The cyclic stability of a Zn||MnHCF cell based on PAM hydrogel at a rate of 5 C.

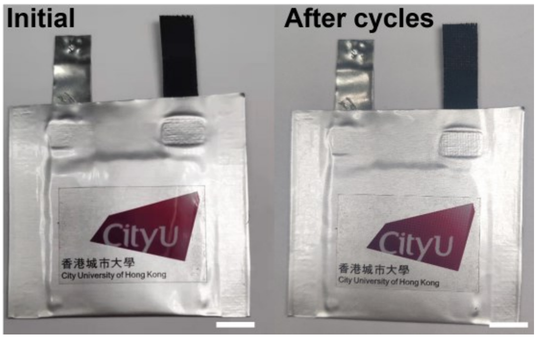

Supplementary Figure 29. The optical pictures of the pouch cell at initial state and after cycling, indicating no swelling (scale bar: 1cm).

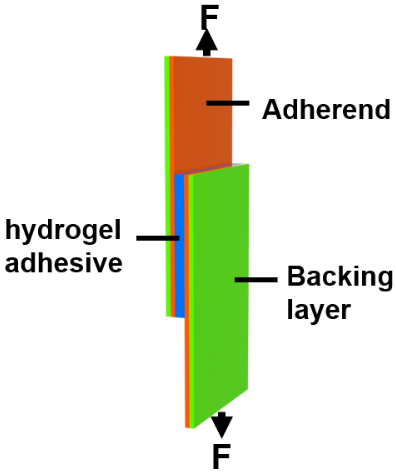

Supplementary Figure 30. The diagram of the lap-shear test.

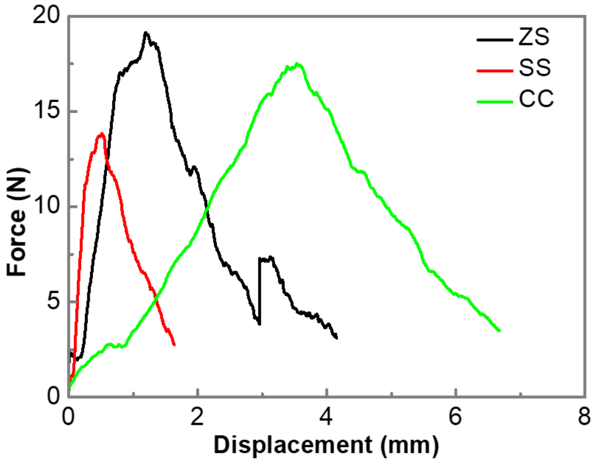

Supplementary Figure 31. Lap-shear tests of ZIG-20 wt% to various substrates.

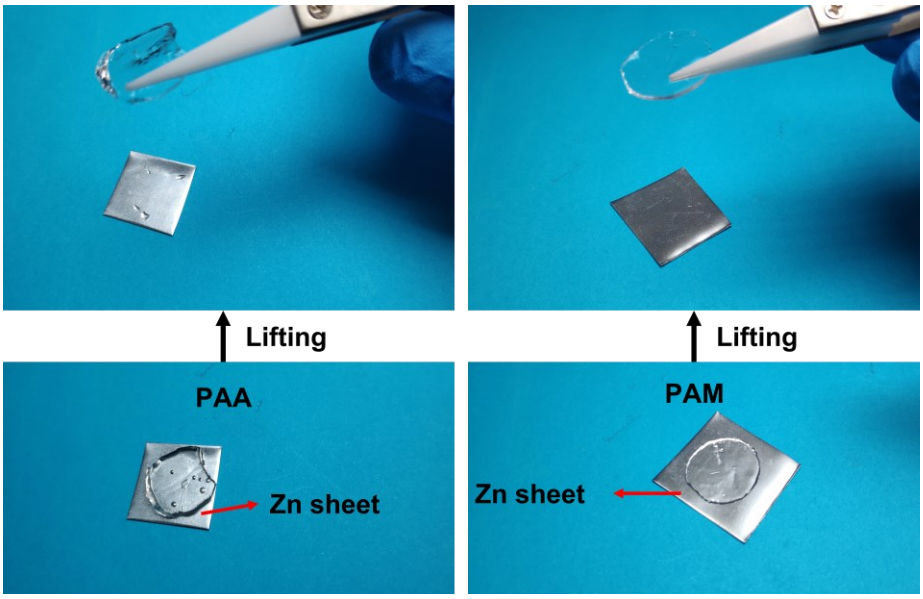

Supplementary Figure 32. Comparison between the adhesiveness of PAA and PAM hydrogels on Zn sheet.

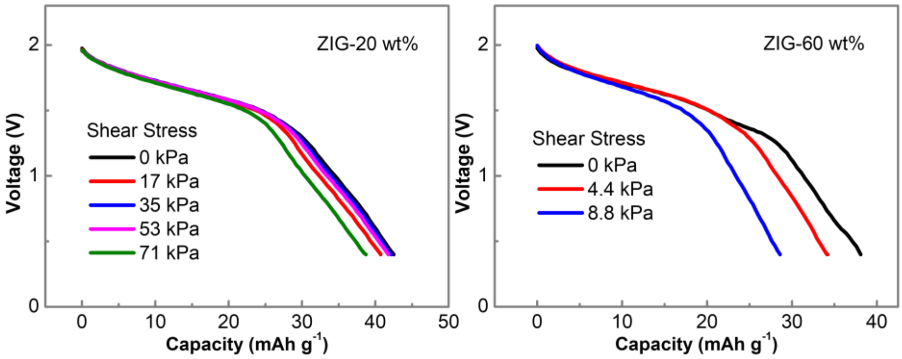

Supplementary Figure 33. The discharge curves of full battery based on ZIG-20 wt% and ZIG-60 wt% electrolytes under shear stress.

Supplementary Table 1. Performance comparison of galvanostatic Zn plating/stripping with different electrolytes in Zn||Zn cells.

Category	Material	Current density (mA cm^{-2})	Areal capacity (mAh cm^{-2})	Cycle life	Voltage hysteresis (mV)	Reference
Lean Water	ZIG	1	0.5	900 h	~120	This work
HPE	GG/SA/EG	0.2	0.2	200 h	40	14
HPE	Self-healable hydrogel	0.1	0.1	550 h	60	15
HPE	SIHE	0.25	0.125	400 h	100	16
HPE	ZL-PAAm	1	1	400 h	80	17
HPE	ZSC	0.5	0.5	200 h	85	18
HPE	CSAM-C	1	1	700 h	-	19

HPE	PVA/Zn(CF_3SO_3)$_2$	0.1	0.1	800 h	100	20
HPE	GAME	0.5	0.5	400 h	~100	21
HPE	Me56	1	1	500 h	50	22
HPE	ZIS-PVA	2	2	200 h	70	23
HPE	PAM/Gelatin/DMAPS	1	1	400 h	180	24
HPE	Zn(Ac)$_2$	2	2	550 h	~90	25
HPE	PAAm/agar/Zn(CF_3SO_3)$_2$	1	1	200 h	~120	26
HPE	PPCu1C-ZMIL5	1	2	500 h	~80	27
HPE	PASHE	2	2	400 h	~100	28
HPE	TRHE	5	1	120 h	~140	29
HPE	Zn^{2+}-CS/PAAM	0.5	0.5	375 h	~110	30
HPE	MMT-PAM	0.5	0.25	300 h	~80	37
SPE	HCPE152	0.02	0.02	1200 h	~100	31
SPE	MSZC	0.05	0.05	700 h	~300	32
SPE	ZCE	0.01	0.005	4000 h	~20	33
SPE	PPM-SPE	0.05	0.025	400 h	~100	34
SPE	PZB-931	0.2	0.1	2500 h	~2.5	35
SPE	WZM	0.1	0.1	360 h	~110	36

Supplementary Table 2. Performance comparison between different electrolytes in Zn||Cu cells.

Category	Material	Current density (mA cm^{-2})	Areal capacity (mAh cm^{-2})	Cycle life	Reference
Lean Water	ZIG	3	3	600 h	This work
		1	3	660 h	
HPE	Zn(Ac)$_2$	0.25	0.5	80 h	25
HPE	PAAm/agar/Zn(CF_3SO_3)$_2$	4	1	50 h	26
HPE	PPCu1C-ZMIL5	1	1	400 h	27
HPE	PASHE	4	1	350 h	28
HPE	TRHE	1	0.5	500 h	29
HPE	Zn^{2+}-CS/PAAM	5	2.5	400 h	30
HPE	MMT-PAM	0.5	0.25	400 h	37

Supplementary references:

1. Qiao L, *et al.* Stable non-corrosive sulfonimide salt for 4-V-class lithium metal batteries. *Nat. Mater.* **21**, 455-462 (2022).
2. Ding. M, *et al.* Change of Conductivity with Salt Content, Solvent Composition, and Temperature for Electrolytes of LiPF6 in Ethylene Carbonate-Ethyl Methyl Carbonate. *J. Electrochem. Soc.* **148**, A1196 (2001).
3. Dion. H, *et al.* Liquid electrolyte development for low-temperature lithium-ion batteries. *Energy Environ. Sci.* **15**, 550-578 (2022).

4. Kyle. D, *et al.* Promising Routes to a High Li+ Transference Number Electrolyte for Lithium Ion Batteries. *ACS Energy Lett.* **11**, 2563-2575 (2017).
5. Yuki. K, *et al.* High-power all-solid-state batteries using sulfide superionic conductors. *Nat. Energy.* **1**, 16030 (2016).
6. Noriaki K, *et al.* A lithium superionic conductor. *Nat. Mater.* **10**, 682-686 (2011).
7. Kyungho Y, *et al.* Challenges and Strategies towards Practically Feasible Solid-State Lithium Metal Batteries. *Adv. Mater.* **34**, 2104666 (2022).
8. Liu S, *et al.* Filler-Integrated Composite Polymer Electrolyte for Solid-State Lithium Batteries. *Adv. Mater.* **1** 2110423 (2022).
9. Nyalaliska W, *et al.* Structure and Evolution of Quasi-Solid-State Hybrid Electrolytes Formed Inside Electrochemical Cell. *Adv. Mater.* **34**, 2110333 (2022).
10. Liu Y, *et al.* Development of quasi-solid-state anode-free high-energy lithium sulfide-based batteries. *Nat. Commun.* **13**, 4415 (2022).
11. Xie J, *et al.* Molecular crowding electrolytes for high-voltage aqueous batteries. *Nat. Energy.* **19**, 1006-1011 (2020).
12. Gao S, *et al.* High-Energy and Stable Subfreezing Aqueous Zn-MnO2 Batteries with Selective and Pseudocapacitive Zn-Ion Insertion in MnO2. *Adv. Mater.* **34**, 2201510 (2022).
13. Dong H, *et al.* Insights on Flexible Zinc-Ion Batteries from Lab Research to Commercialization. *Adv. Mater.* **33**, 2007548 (2021).
14. Wang J, *et al.* Flexible and anti-freezing zinc-ion batteries using a guar-gum/sodium-alginate/ethylene-glycol hydrogel electrolyte. *Energy Storge Mater.* **41**, 599-605 (2021).
15. Li Q, *et al.* Self-Healable Hydrogel Electrolyte toward High-Performance and Reliable Quasi-Solid-State Zn−MnO2 Batteries. *ACS Appl. Mater. Interfaces.* **11**, 38762-38770 (2019).
16. Chan C, *et al.* Single-Ion Conducting Double-Network Hydrogel Electrolytes for Long Cycling Zinc-Ion Batteries. *ACS Appl. Mater. Interfaces.* **13**, 30594-30602 (2021).
17. Zhu M, *et al.* Antifreezing Hydrogel with High Zinc Reversibility for Flexible and Durable Aqueous Batteries by Cooperative Hydrated Cations. *Adv. Funct. Mater.* **30**, 1907218 (2020).
18. Mo F, *et al.* Zwitterionic Sulfobetaine Hydrogel Electrolyte Building Separated Positive/Negative Ion Migration Channels for Aqueous Zn-MnO2 Batteries with Superior Rate Capabilities. *Adv. Energy Mater.* **10**, 2000035 (2020).
19. Huang S, *et al.* Antifreezing Hydrogel Electrolyte with Ternary Hydrogen Bonding for High-Performance Zinc-Ion Batteries. *Adv. Mater.* **34**, 2110140 (2022).
20. Huang S, *et al.* A Self-Healing Integrated All-in-One Zinc-Ion Battery. *Angew. Chem. Int. Ed.* **131**, 4357 -4361 (2019).
21. Lu Y, *et al.* A Semisolid Electrolyte for Flexible Zn-Ion Batteries. *ACS Appl. Energy Mater.* **2**, 6904−6910 (2019).
22. Xu W, *et al.* A cellulose nanofiber–polyacrylamide hydrogel based on a co-electrolyte system for solid-state zinc ion batteries to operate at extremely cold temperatures. *J. Mater. Chem. A.* **9**, 25651–25662 (2021).
23. Li L, *et al.* High-performance dual-ion Zn batteries enabled by a polyzwitterionic hydrogel electrolyte with regulated anion/cation transport and suppressed Zn dendrite growth. *J. Mater. Chem. A.* **9**, 24325–24335 (2021).
24. Qiu M, *et al.* Zwitterionic triple-network hydrogel electrolyte for advanced flexible zinc ion batteries. *Composites Commun.* **28**, 100942 (2021).
25. Shang W, *et al.* Z Establishing High-Performance Quasi-Solid Zn/I2 Batteries with Alginate-Based Hydrogel Electrolytes. *ACS Appl. Mater. Interfaces.* **13**, 24756−24764 (2021).
26. Ji C, *et al.* Natural Polysaccharide Strengthened Hydrogel Electrolyte and Biopolymer Derived Carbon for

Durable Aqueous Zinc Ion Storage. *ACS Appl. Mater. Interfaces.* **14**, 23452−23464 (2022).

27. Hu J, *et al.* Enhancing the Kinetics of Zinc Ion Deposition by Catalytic Ion in Polymer Electrolytes for Advanced Zn–MnO2 Batteries. *Adv. Funct. Mater.* **28**, 2209463 (2022).
28. Zhang W, *et al.* Kinetics-Boosted Effect Enabled by Zwitterionic Hydrogel Electrolyte for Highly Reversible Zinc Anode in Zinc-Ion Hybrid Micro-Supercapacitors. *Adv. Energy Mater.* **12**, 2202219 (2022).
29. Meng Y, *et al.* Developing Thermoregulatory Hydrogel Electrolyte to Overcome Thermal Runaway in Zinc-Ion Batteries. *Adv. Funct. Mater.* **32**, 2206653 (2022).
30. Liu Y, *et al.* Soaking-free and self-healing hydrogel for wearable zinc-ion batteries. *Chem. Eng. J.* **452**, 139605 (2023).
31. Liu G, *et al.* Heteroleptic Coordination Polymer Electrolytes Initiated by Lewis-Acidic Eutectics for Solid Zinc–Metal Batteries. *Chem. Mater.* **34**, 8975–8986 (2022).
32. Wang J, *et al.* Room-temperature fast zinc-ion conduction in molecule-flexible solids. *Mater. Today Energy.* **20**, 100630 (2021).
33. Qiu H, *et al.* Eutectic Crystallization Activates Solid-State Zinc-Ion Conduction. *Angew. Chem. Int. Ed.* **61**, e202113086 (2022).
34. Zhao Z, *et al.* In-situ formed all-amorphous poly (ethylene oxide)-based electrolytes enabling solid-state Zn electrochemistry. *Chem. Eng. J.* **417**, 128096 (2021).
35. Wang M, *et al.* Biomimetic Solid-State Zn2+ Electrolyte for Corrugated Structural Batteries. *ACS Nano.* **13**, 1107−1115 (2019).
36. Wang M, *et al.* A MOF-based single-ion Zn2+ solid electrolyte leading to dendrite-free rechargeable Zn batteries. *Nano Energy.* **56**, 92−99 (2019).
37. Ji S, *et al.* A mechanically durable hybrid hydrogel electrolyte developed by controllable accelerated polymerization mechanism towards reliable aqueous zinc-ion battery. *Energy Storage Mater.* (2022).
